# Comprehensive metabolomics unveil the discriminatory metabolites of some Mediterranean Sea marine algae in relation to their cytotoxic activities

**DOI:** 10.1038/s41598-022-12265-7

**Published:** 2022-05-16

**Authors:** Dina S. Ghallab, Eman Shawky, Reham S. Ibrahim, Mohamed M. Mohyeldin

**Affiliations:** Department of Pharmacognosy, Faculty of Pharmacy, Alexandria University, Alkhartoom Square, Alexandria, 21521 Egypt

**Keywords:** Metabolomics, Natural products, Small molecules

## Abstract

Marine algae have served as a treasure trove of structurally variable and biologically active metabolites. The present study emphasizes on UPLC–MS metabolites fingerprinting for the first systematic broad scale metabolites characterization of three different phyla of marine seaweeds; *Ulva fasciata*, *Pterocladia capillacea* and *Sargassum hornschuchii* along with *Spirulina platensis* harvested from the Mediterranean Sea. A total of 85 metabolites belonging to various classes including mostly fatty acids and their derivatives, terpenoids, amino acids and dipeptides with considerable amounts of polyphenolic compounds. OPLS-DA model offered a better overview of phylum-based discrimination rapidly uncovering the compositional heterogeneity in metabolite profiles of algae extracts. An OPLS model was constructed using the cytotoxic activities against PC3 and MDA-MB-231 tumor cells to succinctly screen cytotoxic discriminatory metabolites among the tested algae species*.* The coefficient plot revealed that unsaturated fatty acids as stearidonic acid and linolenic acid, terpenoids namely as rosmanol, campestanol, dipeptides primarily glutamylglycine, glycyltyrosine along with polyphenolic compounds being abundantly present in *S. platensis* and *U. fasciata* samples with relatively marked cytotoxic potential might be the significant contributors synergistically meditating their anti-proliferative activity against PC3 and MDA-MB-231 tumor cells. Such results serve as baseline for understanding the chemistry of these species and performing strict correlation between metabolite and activity where a lack of information in this regard is observed.

## Introduction

Edible marine seaweeds and microalgae are known to possess tremendous nutritional value and thus regularly consumed as an essential part of traditional Asian diet correcting nutritional deficiencies and promoting health consequences particularly lower incidence of life-threatening chronic diseases like coronary heart disease, diabetes and cancer^1^.

Macroalgae can be broadly categorized into three main taxa based on their morphological pigmentations: Phaeophyta (brown algae), Rhodophyta (red algae) and Chlorophyta (green algae)^2^. Contrary to macroalgae, microalgae are microscopic organisms classified into blue-green algae (Cyanobacteria), dinoflagellates (Dinophyceae) and diatoms (Bacillariophyta)^3^.

Indeed, marine algae have served as a treasure trove of structurally variable and biologically active metabolites including polyphenolic compounds, sulfated polysaccharides, fatty acids, steroids, carotenoids, bioactive peptides, minerals and a plenty of others^4^. Many of these phytochemicals possess unique chemical structure and functions which have been never found in the terrestrial counterparts. This is thought to be due chiefly to distinctive evolutionary features and life in a rather harsh environment^5^.

Diverse classes of unique metabolites have revealed several remarkable biological functionalities such as antioxidant, anticancer, antihypertensive, antihyperlipidemic, anticoagulant, anti-inflammatory, antidiabetic, antifungal, immunomodulatory, neuroprotective, and tissue healing activities^6^. Among all, the anticancer activity of compounds sourced from seaweed presents one of the largest bioprospecting areas in marine natural products.

Egypt which occupies a strategic geographical location straddling both the Mediterranean and the Red Sea, the two main enclosed coastal seas in the world, is considered one of the major producers of the marine flora in the Middle East^7^. In spite of enormous resources enriched with pharmacologically active chemical entities, the studies of seaweeds in Egypt are sparse and scattered.

Since cancer remains one of the premature deadliest diseases globally and its incidence rate keeps rising every year even with the current treatment modalities, the scientific community has focused more interest on new drug discovery strategies to fight against this worldwide health obstacle. Consequently, marine algae have been designated as continuous supply of preparatory models for novel analogues with promising immune-enhancing and anticancer properties. In alignment with earlier scientific investigations concerning cytotoxic potential of marine algae, green macroalga *Ulva fasciata Delile* displayed triggering cytotoxicity in hepatocellular (HepG2) tumor cells via inducing apoptosis and mitochondrial damage^8^. Also, the lipid extracts of two Egyptian marine algae species; *Laurencia popillose* and *Galaxoura cylindrie* were evaluated for their antitumor activity against human breast carcinoma (MCF-7) and hepato carcinoma cells (HEPG2) deciphering potent inhibitory activity^9^.

The present study emphasizes on an area not comprehensively discussed previously, our present study successfully employed UPLC–MS metabolites fingerprinting technique for the first systematic broad scale metabolites characterization of three different phyla (genera) of marine seaweeds; *Ulva fasciata*, *Pterocladia capillacea* and *Sargassum hornschuchii* along with a well-known cyanobacterium, *Spirulina platensis* harvested from the coastal areas in North-Egypt. Considering the staggering complexity of the acquired MS-derived datasets, data mining algorithms seem indispensable. Hence, a number of innovative multivariate statistical analyses were successfully utilized to deal effectively with such massive information where hierarchical cluster analysis heat map was initially done to explore an informative preliminary look at the dataset structure and relationships between algal samples when within-sample variation is sufficiently less than between-sample variation. Therefore, supervised forms of discriminant analysis like Orthogonal Projection to Latent Structures discriminant analysis (OPLS-DA) was extensively utilized for UPLC–MS data analysis to effectively illuminate compositional heterogeneity among algal samples in the context of phylum type. Also, the identified marine algal extracts were specifically screened for their cytotoxic effects, using 3-(4,5-dimethylthiazol-2-yl)-2,5-diphenyltetrazolium bromide (MTT) assay, against the cell proliferation of breast cancer and prostate cancer cell lines in vitro. On the meantime, a supervised classification model Orthogonal Projection to Latent Structures (OPLS) was successfully conducted with a common end goal to present chemical based evidence for algae cytotoxic potential pinpointing precious natural product classes associated with their antitumor properties to be utilized for future generations.

## Results and discussion

### Metabolic profiling of marine algal species

The external chemosphere of marine algae in Egypt has remained overlooked until recent emergence of UPLC–MS-based metabolite profiling approaches. In this regard, the major objective of the current study was to detect, structurally characterize and quantify all secondary metabolites existing in the four marine algal species studied herein in an untargeted, holistic perspective in the context of its genetic diversity and geographical origin in order to get specific cross-comparative analysis and set a framework for its metabolite pattern-based taxonomic classification. To accomplish such goal, UPLC–MS metabolomics approach was successfully conducted.

**Figure 1:**
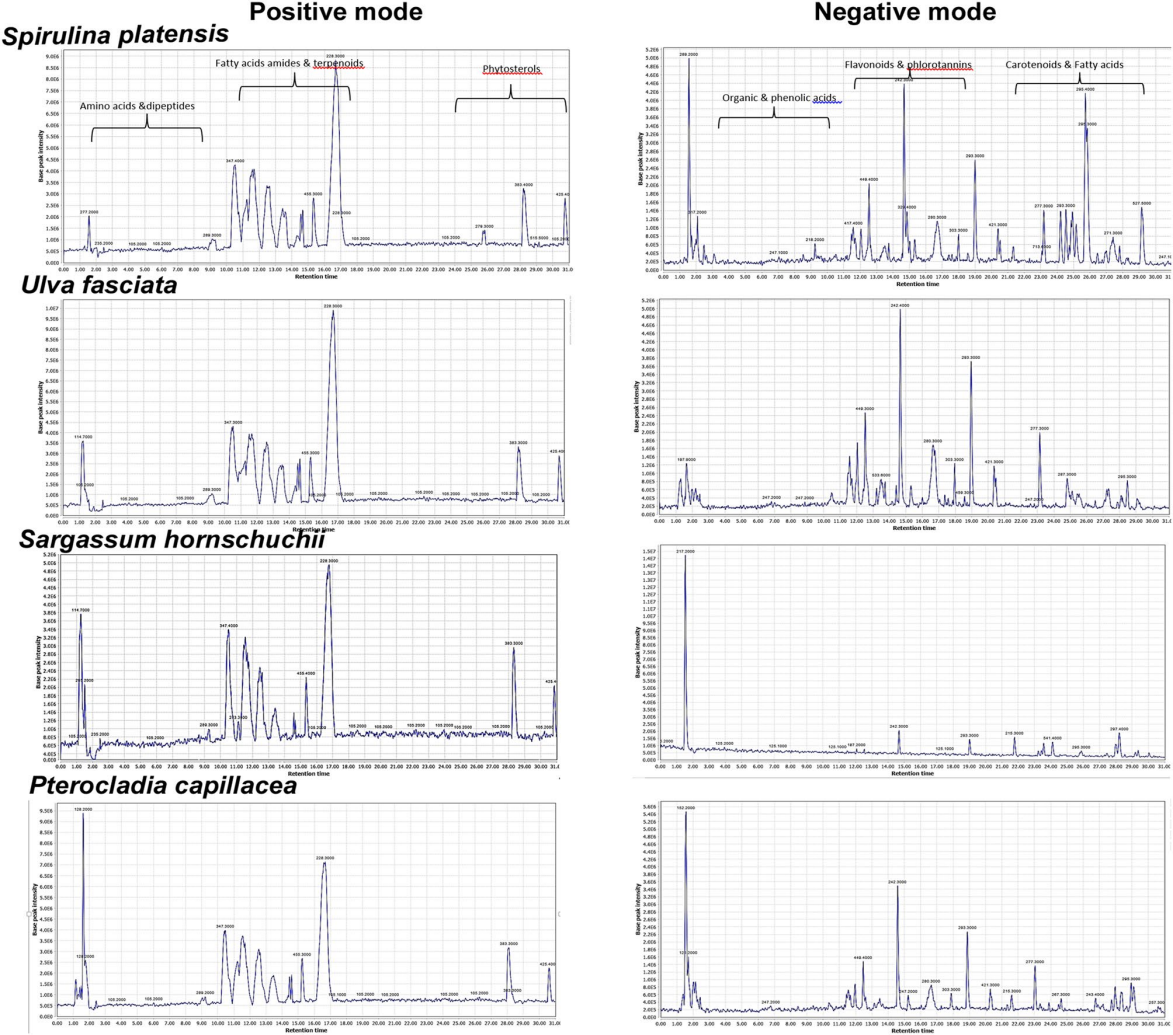
Representative UPLC–MS base peak chromatograms (BPC) collected from the extracts of (**A/A′**) *Spirulina platensis*, (**B/B′**) *Ulva fasciata*, (**C/C**′) *Sargassum hornschuchii* and (**D/D′**) *Pterocladia capillacea* in both positive and negative electrospray ionization modes, respectively.

A total of 85 biochemical compounds satisfactorily retained and baseline separated were tentatively identified from the different seaweed extracts analyzed, which could be broadly categorized into diverse phytochemical classes including fatty acids and their derivatives, flavonoids, phenolic acid, phlorotannins, carotenoids, amino acids and dipeptides (Table 1). Notably, the proportion of detected metabolites within particular categories significantly varied among the four algal species as obviously presented in base peak chromatograms collected in both positive and negative ionization modes of the studied algal samples (Fig. 1). The algal extracts were analyzed in both positive and negative ionization modes providing a comprehensive view of wide array of metabolites. Metabolites were eluted in a decreasing order of polarity, where the first half of the chromatogram accounted for peaks mainly belonging to amino and phenolic acids as well as flavonoidal glycosides followed by flavonoid, triterpene, carotenoids and lastly fatty acids.

**Table 1:** Metabolites identified in algal samples extracts using UPLC-ESI–MS/MS in both negative and positive ionization modes.

No.	Rt (min.)	Identified compounds	Precursor ions	Molecular formula	MS/MS product ions	Chemical class
1	1.03	6-phosphogluconate	277.2 [M + H]^+^	C_6_H_13_O_10_P	183–139–121	Sugars
2	1.16	Methylcitrate	205.3 [M − H]^−^	C_7_H_10_O_7_	191–173–111	Organic acids
3	1.25	Glycerol	114.75 [M + Na]^+^	C_3_H_8_O_3_	74–63–44	Alcohols
4	1.36	Glycerin triacetate	217.25 [M − H]^−^	C_9_H_14_O_6_	174–131–88–70	Alcohols
5	1.42	3-Hydroxybutyrate	105.2 [M + H]^+^	C_4_H_8_O_3_	87–63	Organic acids
6	1.49	Malonic acid	103.34 [M − H]^−^	C_3_H_4_O_4_	59	Organic acids
7	1.55	Cysteine sulfinic acid	152.12 [M − H]^−^	C_3_H_7_NO_4_S	138–110–88	Amino acids
8	1.6	4-Hydroxybenzoic acid 4-O-glucoside	299.2 [M − H]^−^	C_13_H_16_O_8_	137–93	Hydroxybenzoic acids
9	1.64	Valine	118.35 [M + H]^+^	C_5_H_11_NO_2_	100–72–55	Amino acids
10	1.69	Malic acid	133.12 [M − H]^−^	C_4_H_5_O_5_	115	Organic acids
11	1.74	2-Hydroxyglutaric acid	147.23 [M − H]^−^	C_5_H_8_O_5_	103–59	Organic acids
12	1.82	Methyladenosine	282.25 [M + H]^+^	C_11_H_15_N_5_O_4_	146–132–116–106	Nucleotides
13	1.87	Succinic acid	117.21 [M − H]^−^	C_4_H_6_O_4_	73	Organic acids
14	1.94	Glutamylglycine	205.23 [M + H]^+^	C_7_H_12_N_2_O_5_	75–58	Dipeptides
15	2.01	Isoferulic acid 3-sulfate	273.15 [M − H]^−^	C_10_H_10_O_7_S	193–149–178	Phenolic acids
16	2.07	*p*-Hydroxybenzoic acid	137.12 [M − H]^−^	C_7_H_6_O_3_	93	Hydroxybenzoic acids
17	2.10	Syringic acid	197.15 [M − H]^−^	C_9_H_9_O_5_	182–153–167	Phenolic acids
18	2.21	Acetyl pyrrolidone	128.14 [M + H]^+^	C_6_H_9_NO_2_	85–43	Nitrogenous compounds
19	2.26	Phloroglucinol	125.15 [M − H]^−^	C_6_H_5_O_3_	97	Phenolic compounds
20	2.35	Caffeoylglycerol	253.2 [M − H]^−^	C_12_H_14_O_6_	179	Phenolic glycerides
21	2.51	Ellagic acid	301.2 [M − H]^−^	C_14_H_6_O_8_	257–229–185	Hydrolyzable tannins
22	2.67	Trimethyl citrate	235.15 [M + H]^+^	C_9_H_14_O_7_	191–173–111	Organic acids
23	3.5	Catechin	289.2 [M − H]^−^	C_15_H_14_O_6_	245–205–139	Flavonoids
24	4.45	Acetylornithine	173.12 [M − H]^−^	C_7_H_14_N_2_O_3_	131–116-115–70	Amino acids
25	5.23	Glutamylcysteine	251.35 [M + H]^+^	C_8_H_14_N_2_O_5_S	121–104–86	Dipeptides
26	5.44	Phenylpyruvic acid	163.15 [M − H]^−^	C_9_H_8_O_3_	144–116–88	Organic acids
27	5.94	Brevifolin	247.2 [M − H]^−^	C_12_H_8_O_6_	219–191–163	Alkyl-phenylketones
28	6.21	2-Isopropylmalate	175.2 [M − H]^−^	C_7_H_12_O_5_	157–115-85	Organic acids
29	6.8	Hydroxyferulic acid	209.2 [M − H]^−^	C_10_H_10_O_5_	193–177–149	Phenolic acids
30	8.44	Pantothenic acid	218.2 [M − H]^−^	C_9_H_17_NO_5_	200–182–174	B vitamins
31	8.75	Rosmarinic acid	359.42 [M − H]^−^	C_18_H_16_O_8_	179–137–143	Phenolic acids
32	9.21	Tetrahydroxyisoflavanone	289.3[M + H]^+^	C_15_H_12_O_6_	269, 259–136	Flavonoids
33	9.85	Caffeic acid isoprenyl ester	247.15 [M − H]^−^	C_14_H_16_O_4_	179–135	Phenolic acids
34	10.5	Phloroglucinol dimer derivative	517.11 [M − H]^−^	…….	247–125	Phlorotannins
35	11.25	Glycyltyrosine	239.15 [M + H]^+^	C_11_H_14_N_2_O_4_	165–119	Dipeptides
36	11.56	Kaempferol*-*O-pentose	417.42 [M − H]^−^	C_20_H_19_O_11_	285–257–151	Flavonoids
37	11.67	3′-*O*-Methylequol	273.25 [M + H]^+^	C_16_H_16_O_4_	147–123	Flavonoids
38	11.89	Naringenin pentose	405.35 [M + H]^+^	C_20_H_21_O_10_	273–153–121	Flavonoids
39	12.14	Kaempferol-methylether-*O*-glucoside	463.35 [M + H]^+^	C_22_H_22_O_11_	301–286–258–153	Flavonoids
40	12.25	Hydroxydecanoic acid	187.2 [M − H]^−^	C_10_H_20_O_3_	143–125	Fatty acids
41	12.55	Taxifolin-O-rhamnoside	449.35 [M − H]^−^	C_21_H_22_O_11_	303–285–175–125	Flavonoids
42	12.63	Rosmanol	347.35 [M + H]^+^	C_20_H_26_O_5_	301–231	Terpenoids
43	12.87	Dimethoxy-luteolin-glucoside	475.22 [M − H]^−^	C_23_H_23_O_11_	315–285-241–179-151	Flavonoids
44	13.2	Kaempferol-3-O-malonylglucoside	533.15 [M − H]^−^	C_24_H_22_O_14_	447–285–241–151	Flavonoids
45	13.53	Cypellocarpin C	521.5 [M + H]^+^	C_26_H_32_O_11_	337–189	Phenol glycosides
46	13.66	8-Pentadecenal	225.25 [M + H]^+^	C_15_H_28_O	207–197	Fatty aldehydes
47	13.72	Quercetin	301.15 [M − H]^−^	C_15_H_10_O_7_	179–151	Flavonoids
48	14.68	Lauramide	200.12 [M + H]^+^	C_12_H_25_NO	156	Fatty acid amides
49	14.87	Hydroxy myristamide	244.21 [M + H]^+^	C_14_H_29_NO2	226–199–181	Fatty acid amides
50	15.33	Oleanonic acid	455.15 [M + H]^+^	C30H47O3	437–409–395	Terpenoids
51	15.8	1-Palmitoyl-GPA (16:0)	409.35 [M + H]^+^	C_19_H_37_O_7_P^-2^	256–212–171–153	Glycerolphospholipids
52	16.52	Glycitein 7-O-glucuronide	459.45 [M − H]^−^	C_22_H_20_O_11_	283, 268, 224, 133	Flavonoids
53	16.69	Myristamide	228.25 [M + H]^+^	C_14_H_29_NO	201	Fatty acid amides
54	17.4	Taxifolin	303.25 [M − H]^−^	C_15_H_12_O_7_	285–175–125	Flavonoids
55	17.77	Apigenin	269.22 [M − H]^−^	C_15_H_10_O_5_	255–165–151	Flavonoids
56	18.9	Quercetin dimethylether	329.35 [M − H]^−^	C_17_H_14_O_7_	315–300–179–151	Flavonoids
57	20.04	Fucophlorethol A	373.12 [M − H]^−^	C_18_H_14_O_9_	247–229	Phlorotannins
58	20.34	Hydroxylauric acid	215.25 [M − H]^−^	**C**_**12**_**H**_**24**_**O**_**3**_	171–153	Fatty acids
59	20.58	Hydroxylinolenic acid	293.3 [M − H]^−^	C_18_H_29_O_3_	275–231	Fatty acids
60	20.97	Pentadecenoic acid	239.3 [M − H]^−^	C_15_H_28_O_2_	195–193–155	Fatty acids
61	21.33	C16: 1/C20: 5 phosphatidylglycerol	765.15 [M − H]^−^	C_42_H_71_O_10_P	511–463–529–481–253	Glycerolphospholipids
62	23.13	Tetrafuhalol A	529.22 [M − H]^−^	C_24_H_18_O_14_	387	Phlorotannins
63	23.9	Eckol derivative	541.12 [M − H]^−^	……	371–229	Phlorotannins
64	24.12	Carnosic acid	331.3 [M − H]^−^	C_20_H_28_O_4_	278–244	Terpenoids
65	24.3	Stearidonic acid	277.3 [M + H]^+^	C_18_H_28_O_2_	255–233–162	Fatty acids
66	24.44	Linolenic acid	277.15 [M − H]^−^	C_18_H_30_O_2_	233–111–69	Fatty acids
67	24.58	Dihydroxypalmitic acid	287.25 [M − H]^−^	C_16_H_34_O_4_	269–251–207	Fatty acids
68	24.7	C18: 3 /C13:0 phosphatidylglycerol	701.12 [M − H]^−^	C_37_H_67_O_10_P	424–442–277–505–487	Glycerolphospholipids
69	24.87	Echinenone	549.4 [M − H]^−^	C_40_H_54_O	531–203	Carotenoids
70	24.98	Palmitoleic acid methyl ester	267.3 [M − H]^−^	C_17_H_32_O_2_	253–141	Fatty acid esters
71	25.7	Hydroxylinoleic acid	295.22 [M − H]^−^	C_18_H_32_O_3_	277–233–59	Fatty acids
72	25.9	Eicosapentaenoic acid	301.29 [M − H]^−^	C_20_H_30_O_2_	257–108	Fatty acids
73	26.5	Fucoxanthinol	615.25 [M − H]^−^	C_40_H_58_O_5_	597–579–147	Carotenoids
74	26.8	Hydroxymyristic acid	243.4 [M − H]^−^	C_14_H_28_O_3_	225–181	Fatty acids
75	27.32	Linoleic acid methyl ester	293.35 [M − H]^−^	C_19_H_34_O_2_	277–233–111–59	Fatty acid esters
76	27.72	Diadinoxanthin	581.2 [M − H]^−^	C_40_H_54_O_2_	563–545–221	Carotenoids
77	28.11	Hydroxypalmitic acid	271.35 [M − H]^−^	C_16_H_31_O_3_	253–209	Fatty acids
78	28.3	Campesterol	401.6 [M + H]^+^	C_28_H_48_O	383–268–147–161	Phytosterols
79	28.45	Hydroxystearic acid	299.3 [M − H]^−^	C_18_H_36_O_3_	281–237	Fatty acids
80	28.6	Oleic acid	281.2[M − H]^−^	C_18_H_34_O_2_	237–124–59	Fatty acids
81	28.75	Nonadecenoic acid	295.4 [M − H]^−^	C_19_H_36_O_2_	251–128	Fatty acids
82	29.42	Nonadecanoic acid	297.23 [M − H]^−^	C_19_H_38_O_2_	253	Fatty acids
83	29.8	Campestanol	425.6 [M + Na]^+^	C_28_H_50_O	385–149–163	Phytosterols
84	30.03	Betulinic acid	455.12 [M − H]^−^	C_30_H_48_O_3_	440–435–407	Terpenoids
85	30.3	Methyl stearate	299.2 [M + H]^+^	C_19_H_38_O_2_	284–101–87	Fatty acid esters

Table 1 summarized the complete list of annotated metabolites obtained from the analyzed algal samples along with their structural information including retention time, the precursor ions [M − H]^−^/[M + H]^+^, the characteristic fragment ions and molecular formula. Compounds were numbered according to their elution order. The detailed scheme used to characterize each class of metabolites is given below.

### Identification of phenolic acids

The phenolic acids and their derivatives represented in peaks **(8, 15, 16, 17, 20, 29, 31, 33)** and detected with higher responses in negative ionization mode exhibited a common fragmentation pathway based on a loss of the carboxyl group (CO_2_, − 44 Da)^10^. Compound (**8**), having a precursor ion [M − H]^−^ m/z at 299.2 was tentatively characterized hydroxybenzoic acid 4-O-glucoside. Tandem mass of this peak displayed a predominant ion at m/z 137 mainly attributed to the loss hexosyl moiety (162 Da) from precursor ion which further dissociated furnishing a product ion at m/z 93 after the loss of CO_2_ reinforcing the presence of hydroxybenzoic acid. Compound (**15**) generated a deprotonated [M − H]^−^ ion at m/z 273.15, intense fragments at m/z 193 and 149 corresponding to the loss of SO_3_ (80 Da) and further loss of CO_2_ (44 Da) from the precursor ion, while the product ion at m/z 178 (M − H − 15)^−^ resulted from the loss of one radical CH_3_. All produced ions reinforced the presence of isoferulic acid ion (193 Da) and the compound was reasonably annotated as isoferulic acid 3-sulfate (Table 1).

The proposed candidate for peak (**31**) was rosmarinic acid, a caffeic acid ester, due to the molecular ion at m/z 359.42 and the characteristic product ion of m/z 179 (presence of caffeic acid ion) generated by the hemolytic or heterolytic cleavage of the bond with ester moiety (Table 1). The presence of caffeic acid ion was further confirmed by the diagnostic product ions at m/z 137 [M − H − 44] and m/z 143 [M − H − 36] representing the loss of CO_2_ and two H_2_O units, respectively from the precursor ion.

### Identification of phlorotannins

Phlorotannins constitute inexhaustible class of naturally occurring unique polyphenolic entities derived from the polymerization of the monomer phloroglucinol^11^.

Detection of phlorotannins was observed in both positive and negative ionization modes; however, peaks characterization was much more aided with ESI^**−**^ being more sensitive for phenolics detection, besides their adequate fragmentations easy to interpret.

The suggested compound for peak (**34**) was phloroglucinol dimer derivative with a parent ion of m/z 517.11 accompanied with intense fragment ions of m/z 247 [phloroglucinol dimer-H]^−^ and 125 [phloroglucinol-H]^−^. Such successive losses of phloroglucinol units were suggestive for the core C–O–C linkage between two phloroglucinol monomers. This phloroglucinol oligomer was further confirmed by comparing its mass fragmentation profile with previously reported data^12^.

Tetrafuhalol A was the candidate for peak (**62**) detected at m/z 529.22. By MS/MS analysis of tetrafuhalol formed through C–O–C oxidative coupling of phloroglucinol monomeric units, the main MS^2^ fragments observed were at m/z 387 [M − H]^−^ as a base peak resulting from the loss of a single phloroglucinol molecule that was further fragmented in MS^3^ with the combined loss of two additional phloroglucinol subunits and a water molecule (266 Da = 124 + 124 + 18). As so, compound **66** should correspond to two fuhalol moieties linked by ether bonds and contain an additional OH group in its backbone (Table 1).

### Identification of organic acids

Nine compounds (**2, 5, 6, 10, 11, 13, 22, 26, 28**) identified as organic acids were recorded in the first half of the chromatographic runs with much higher response in negative ionization mode. All exhibited similar characteristic mass fragmentation profiles generated by losses of CO_2_ and H_2_O.

Asides, five dicarboxylic *acids* with respective molecular ions of m/z 103.34, 133.12, 147.23, 117.12 and 175.2 were assigned in peaks **6, 10, 11, 13** and **28** and accordingly annotated as malonic acid, malic acid, hydroxyglutaric acid, succinic acid and 2-isopropylmalic acid, respectively (Table 1).

### Identification of flavonoids

In the present study, a total of 14 flavonoid peaks were recorded mainly in negative polarity mode.

The highly useful fragmentations in terms of flavonoid structural characterization are those established by cleavage of two C–C bonds of the pyran ring, resulting in structurally informative retro-Diels–Alder (RDA) anions [^i,j^A^−^] and [^i,j^B^−^] which provide value-added information on the number and type substituents in A and B rings^13^.

### Identification of flavonols

For quercetin (**47**), the deprotonated ion at m/z 301.15 [M − H]^−^ also corresponded to a base peak. Retrocyclisation pathway for bond 1 furnished two fragment signals with high intensities at m/z 179 [^1,2^A]^−^ and 151 [^1,3^A]^−^.

### Identification of flavones

The proposed flavone for peak **55** was apigenin based on its quasi-molecular ion [M − H]^−^ at m/z 269.22 also acting as base peaks besides the structurally highly informative daughter ion species at m/z 151 [^1,3^A]^−^ and 165 [^1,3^B]^−^ arising from RDA fragmentation mechanisms. Also, fragment ion at m/z 225 was generated by loss of CO_2_ from endocyclic keto group of the C ring.

### Identification of isoflavonoids

3,4,5,7-Tetrahydroxyisoflavanone was proposed as compound (**32**) with a precursor ion [M + H]^+^ m/z of 289.3. Further CID-MS^2^ analysis generated fragment ions of high relative abundance represented by *m/z* 269 and 259 suggestive for neutral loss of H_2_O and CO from the precursor ion, respectively. In addition, a characteristic fragment ion ^1,3^B^+^ represented by *m/z* 136 and produced by a retro-Diels–Alder cleavage was observed. By comparing previous data^14^, with its MS^2^ spectrum we could draw conclusion regarding the identity of compound **32**.

### Identification of flavonoid glycosides

Compound (**36**) presented a [M − H]^−^ ion at m/z 417.42 accompanied with a predominant aglycone base peak at m/z 285 [M − H − 132]—generated through the loss of a pentose moiety. Confirmation of kaempferol as aglycone moiety was gained from MS^2^ product ions at m/z 259 after the loss of CO (28 Da) along with distinctive RDA signal at m/z 151. Based on the above MS^n^ data, compound **36** was accordingly annotated as kaempferol-O-pentose.

The proposed compound for peak **(41**) was taxifolin-O-rhamnoside on the basis of its quasi-molecular ion at m/z 449.35 for [M − H]^−^ and a predominant aglycone base peak at m/z 303 characterized by the loss of neutral rhamnose residue. At high CE (70 eV), ions corresponding to a fragmentation pathway of taxifolin described above were also observed and led to the confident compound identification.

The metabolite at peak **(52**) gave rise to an intense precursor [M − H]^−^ ion at m/z 459.45. Its MS^2^ fragmentation spectrum revealed the presence of a prominent ion at m/z 283 [M − H − 176]^−^ arising from neutral loss of glucuronyl moiety. Glycitein was confirmed as aglycone by the characteristic fragments (m/z 268 and 224) obtained at high CE (70 eV) from consecutive losses of methyl radical and CO_2_. Furthermore, a typical RDA fragment [^1,3^B]^−^ at *m/z* 133 with a high relative abundance was registered and clearly suggested the existence of an extra hydroxyl substituent. Based on the above arguments and the literature data^14^, compound (**52**) was tentatively assigned as glycitein-O-glucuronide.

### Identification of terpenoids, carotenoids and phytosteroids

Under current analysis conditions, only 7 peaks were recorded in terpenoids category including two phenolic diterpenoids (**42, 64**), two pentacyclic triterpenoids (**50, 84**) and three carotenoids (**69, 73, 76**). Such compounds were well clustered in the second half of the chromatograms.

Additionally, information obtained from positive ionization mode of MS/MS spectra revealed the presence of two characteristic peaks (**78, 83**) corresponding to phytosterols as listed in Table 1.

In positive polarity mode, compound **42** presented a molecular ion peak with great intensity at m/z 347.35 for [M + H]^+^. The MS^2^ product ions of m/z 301 and m/z 231 gained at high CE (60 eV) initially came from the combined loss of H_2_O and CO (46 Da) followed by cleavage of molecules pentene, water, and carbon monoxide. According to the precursor and product ions, and further confirmed by reference literature^14^, compound **42** was tentatively established to rosmanol.

Another phenolic abietane-type diterpenoid (**64**) was identified as carnosic acid whose MS data presented a precursor ion at m/z 331.3 which in turn produced a major fragment ion at m/z 278 through loss of CO_2_ being a characteristic fragmentation feature of phenolic acids. Subsequently, this decaboxylated carnosic acid further dissociated and lost a propyl moiety (− 43 Da) forming a peak signal of relatively lower intensity at m/z 244. This fragmentation route was in agreement with previous literature data^12^.

Compounds **50** and **84** displayed structural similarities and both were characterized as pentacyclic triterpenes. For compound **50** showing as precursor ion at [M + H]^+^ at m/z 455.15, fragment ions at m/z 437, 409, 395 generated by successive losses of H_2_O, CO, C_3_H_7_^**.**^ radical led to its identification as oleanonic acid further confirmed by literature data^15^.

The deacetylated metabolite fucoxanthinol was characterized in this study at respective peak **73** with a [M − H]^−^ ion of m/z 615.25 and exhibited MS^2^ fragments at m/z 597 and 579 already interpreted by the sequential loss of two water molecules from the polyene chain. Moreover, the fragment ion with a quite intense level at m/z 147 corresponding to the dehydrated terminal ring after water loss and cleavage at the 7, 8 bond (C_10_H_11_O), was readily recorded.

Campesterol (peak **78**) with a precursor ion at m/z 401.6 accompanied with an abundant product ion also corresponded base peak at m/z 383 consistent with the loss of a water molecule was evidently recorded. Employing higher collision energy (70 eV) led to the formation of dominant fragment ions most likely formed upon the cleavage within the C-ring and partial side chain fragmentation. Scission between C9/C11 and C8/14 of the C-ring led to *m/z* 147 with higher relative abundance while the abundant fragment ion of *m/z* 161 resulted upon scission between C11/C12 and C8/C14.

### Identification of fatty acids

Fatty acids, the least polar metabolites eluted late, overwhelmingly dominated the secondary metabolites in the examined algal samples and readily predominated by hydroxylated (oxylipins) and unsaturated forms.

An unsaturated fatty acid “linolenic acid” (compound **66**) with an observed [M − H]^−^ of m/z 277.15 was recorded. The high-energy spectrum of linolenic acid provided three key product ions at m/z 233, 111 and 69 with the different peak intensities respectively compatible with [M – H–CO_2_]^−^, [M – H–C_10_H_16_O_2_]^−^ and [M – H–C_13_H_20_O_2_]^−^ further verified by consulting literature data^16^.

Three methyl branched fatty acids with representative peaks; **70, 75 and 85** were evidently detected under the current ESI conditions and identified as palmitoleic acid methyl ester, linoleic acid methyl ester and stearic acid methyl ester, respectively. These compounds gave precursor ions of m/z 267.3, 293.35 and 299.2, respectively and shared similar fragmentation behavior. For the sake of clarity, the preliminary source-induced fragmentation with a collisional energy of 40 eV enhanced the generation of demethylated [M − H–CH_3_]^−^ ions diagnostic for methyl branched fatty acids followed by favorable decarboxylation as illustrated in Table 1.

Besides, MS spectra interpretation allowed for detection of seven OHFA candidates in the pooled algae sample with respective peaks (**40, 58, 59, 71, 74, 77, 79**). In details, MS revealed six monohydroxy-fatty acids in negative polarity mode identified as hydroxydecanoic acid (**40**), hydroxylauric acid (**58**), hydroxylinolenic acid (**59**), hydroxylinoleic acid (**71**), hydroxymyristic acid (**74**), hydroxypalmitic acid (**77**) and hydroxystearic acid (**79**) with the precursor ions [M − H]^−^ of 187.2, 215.25, 293.3, 295.22, 243.4, 271.35 and 299.3, respectively. They all produced predominant ions consistent with the neutral loss of CO_2_ (− 44 Da) along with extra loss of water molecule (− 18 Da). In accordance with literature (Gowda et al.^17^), a typical product ion C_2_H_3_O_2_^−^ (m/z 59) corresponding to a McLafferty-type rearrangement evidently manifested by the cleavage of the C2–C3 bond was sometimes recorded (Table 1).

Three fatty acid amides (FAAs) were detected as major peaks in the positive polarity mode being more informative with a better signal-to-noise ratio than negative ionization one for these lipid species^18^. Remarkably, three protonated ions species could be easily recognized as fatty acid amides; lauramide (compound **48**), myristamide (compound **49**) and hydoxymyristamide (compound **53**). Under the relatively low-energy regime of CID, the qualitatively similar fragmentation strategy was applied to all recognized FAAs mainly manifested by accompanying elimination of NH_3_ and CO molecules from the respective parent ions.

### Identification of glycerolphospholipids

MS/MS spectra examination in both ESI^+^ and ESI^−^ allowed the identification of three glycerolphospholipids at respective peaks **51, 61** and **68** (Table 1). In details, the metabolite at peak **51** exhibiting a [M + H]^+^ ion at m/z 409.35 was simply assigned as 1-palmitoyl-GPA (16:0). Its product–ion spectrum of the [M + H]^+^ ion displayed ions at m/z 153 and 171 reflecting neutral loss of 16:0 fatty acyl substituent a free fatty acid and as a ketene, respectively. Further, palmitic acid was confirmed as the bound fatty acid by the characteristic fragment ions at 256 and 212.

In the negative ion mode, PG at peak **61** yielded an abundant [M − H]^−^ ion at m/z 765.15 and was identified as C16:1 /C20:5 phosphatidylglycerol further confirmed by the existing literature^16^. Following the same pathway, two high intensity fragments at m/z 511 and 463, reflecting neutral loss of 16:1 and 20:5 fatty acids at sn-1 and sn-2, respectively were recorded in its MS/MS spectrum. Asides, the mass spectrum contains signals at m/z 529 and 481 attributed to neutral loss of the fatty acyl substituent at sn-1 and sn-2 as a ketene, respectively.

### Identification of amino acids and dipeptides

MS/MS spectra inspection in both ESI^+^ and ESI^−^ permitted the characterization of a total of 3 free amino acids, oxidized and acylated derivatives as well as 3 dipeptides well clustered in the first half of the chromatographic run with greater sensitivity and clearer detection in positive mode (Table 1).

In the beginning of analysis, the highly polar metabolite (peak **9**) exhibiting the respective [M + H]^+^ ion at m/z 118.35 was detected. During MS^2^ analysis, two major signals coherent with series of neutral loss of 18 Da (H_2_O) and 28 Da (CO) followed for intensity by a fragment ion steadily matching with a neutral loss of 17 Da (NH_3_) were recorded. Hence, compound **9** could be unequivocally identified as valine.

An intense signal (**7**) recorded at m/z 152.12 [M − H]^−^ was assigned as cysteine sulfinic acid. To confirm such an assignment, higher collision dissociation energy was employed yielding diagnostic signals at m/z 138 and 110 evidently explained as the sequential loss of NH_3_ and H_2_O. In its CID-MS/MS spectrum, an additional minor signal was detected at m/z 88 and obtained through the unique breaking of sulfur dioxide (− 64 Da) from precursor ion.

For compound **24** with a [M − H]^−^ ion at m/z 173.12, the loss of 43 mass units at low collision energy indicated the presence of an acetyl group and yielded a base peak at m/z 131. Subsequently, informative ions corresponding to the fragmentation pathway of ornithine moiety (116, 115 and 70) respectively assigned as [M – H–NH_3_]^−^, [M – H–H_2_O]^−^ and [M – H–NH_3_–H_2_O–CO]^−^ were also observed. Taken together with previous data (Zhang et al.^19^), compound **24** could be unequivocally identified as acetylornithine (Table 1).

Under current analysis condition, three dipeptides were detected in peaks (**14, 25, 35**) and shared similar fragmentation pathway fundamentally manifested by the concerted cleavage of the protonated amide bond linking two consecutive amino acids expelling N-terminal and C-terminal amino acid fragments^20^.

A peak (**14**) detected at m/z 205.23 [M + H]^+^ was assigned as glutamylglycine. For protonated Glu–Gly, a prominent fragmentation route initiated with a glutamine moiety (− 130 Da) elimination presumably mainly through amide bond cleavage producing an abundant signal at m/z 75 which in turn showed high propensity to lose NH_3_ recording a signal at m/z 58.

### Identification of nucleosides

In the present study, only one nucleoside (Peak **12**) provides a significantly better signal in the positive ESI mode.

Practically speaking, compound (**12**) generated a [M + H]^+^ ion at m/z 282.25, and a base peak at m/z 146 explained by the neutral loss of a ribose moiety (− 134 Da) from its precursor ion. In the MS^2^ spectrum of m/z 146 with CE = 40 eV, the observation of predominant peaks at m/z 132 [M − H–CH_3_]^−^, 116 [M − H–CH_3_–NH_3_]^−^, 106 [M − H–CH_3_^_^HCN]^−^ proved the nucleobase identification as methyl adenine, According to the mass spectrum data obtained accompanied with previous literature data^21^, compound (**12**) was annotated as methyladenosine.

### Relative quantification of the identified metabolites

**Figure 2:**
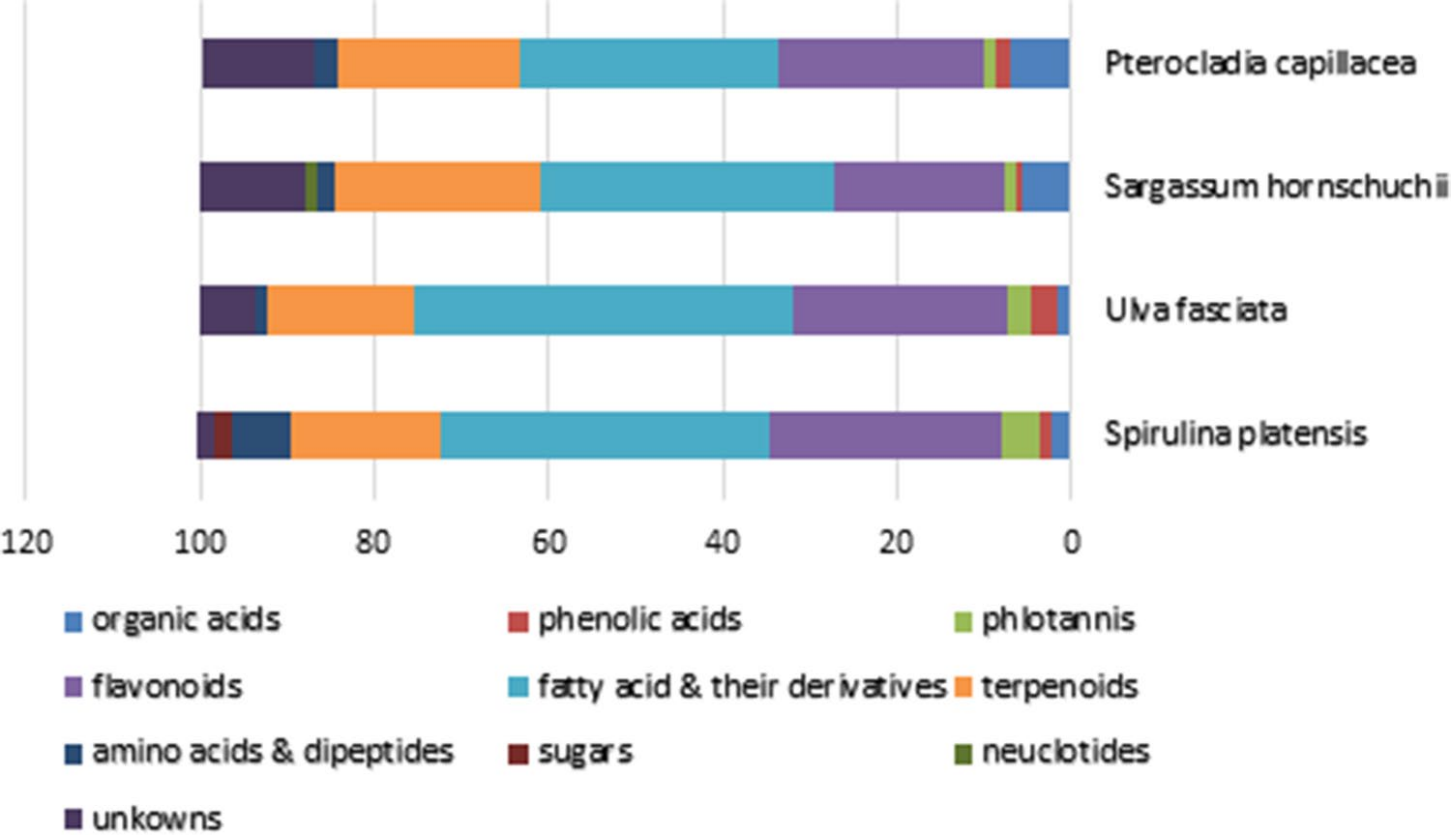
Mean relative percentile levels of the main chemical classes of the four tested algal species expressed as mg standard Equivalents (Eq.)/g dry weight.

Asides, all the identified metabolites were relatively using standard compounds successfully utilized to calculate the relative concentrations of fatty acids and their derivatives, flavonoids, phenolic acid, phlorotannins, organic acids, terpenoids, amino acids and dipeptides (expressed as mg standard Equivalents/g dry extract (DE)) of each tested sample (Table S3). As observed in the stacked bar char (Fig. 2), there was an obvious qualitative and quantitative variation in the chemical composition of the tested algae extracts. As a general trend, fatty acids and their derivatives overwhelmingly dominated the secondary metabolites and were detected in the extracts of all tested samples where the highest relative amount of fatty acids and their derivatives were readily found in *U. fasciata* followed by *S. platensis* and *S. hornschuchii* extracts (Fig. 2). Meanwhile, phenolic constituents dominated the secondary metabolite in the studied algal species except *S. hornschuchii* which exhibited a relatively higher number of fatty acids and their derivatives as well as terpenoids than phenolics. Comparatively, it is noted that the content of amino acids and dipeptides was slightly higher in *S. platensis* and *P. capillacea* than other algae species. Moreover, within the algae species, *S. hornschuchii and P. capillacea* presented relatively higher organic acids content that others as clearly depicted in Fig. 2.

### Chemical profiling of marine algae using UPLC–MS multivariate data analysis

With a view to reveal intrinsic trends or recognition patterns among algae specimens in an unbiased manner, principal component analysis (PCA) was the preferential option rapidly capturing the maximum inherent qualitative and quantitative variability in their chemical features. As depicted in Fig. S1, the PCA score plot representing 74.8% of entire variation in samples offered a considerable classification of the algal samples into two main clusters indicating observable differences in their chemical makeup, where *S. platensis* and *U. fasciata* samples were both clustered along the negative side of PC1 whereas *S. hornschuchii* and *P. capillacea* were relatively clustered altogether on the positive side of PC1.

**Figure 3:**
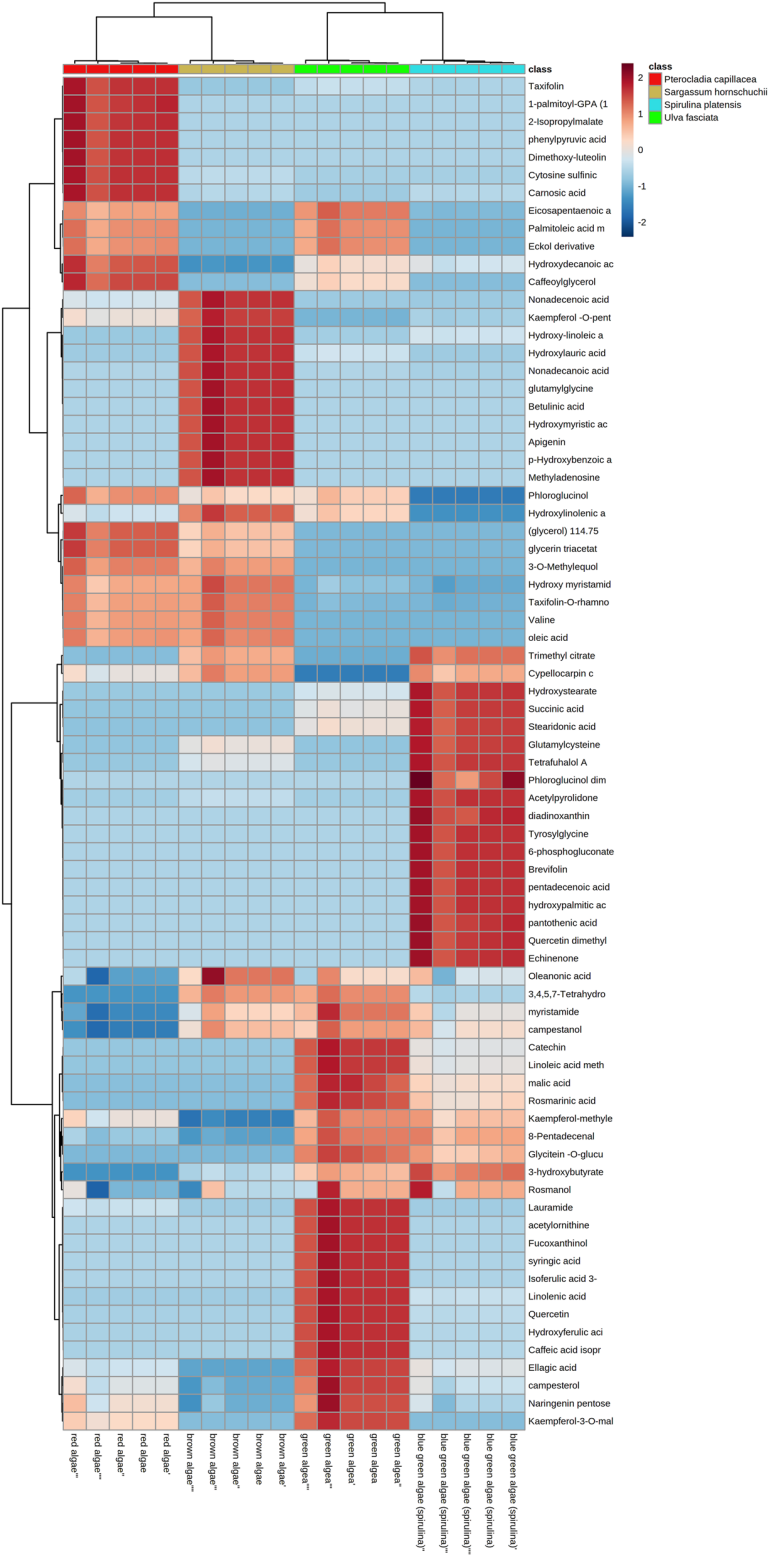
Hierarchical analysis heat maps of all identified metabolites in *S. platensis*, *U. fasciata*, *P. capillacea* and *S. hornschuchii* algae species. Brick red and blue indicate higher and lower abundances, respectively.

Furtherly, the data obtained from UPLC–MS analyses were subjected to unsupervised pattern recognition analysis utilizing the hierarchical cluster analysis (HCA) heat-map to visually present a comprehensive view of the clustering trend of different algae specimens via understandable graphical output data comprising a set of intrinsically weighing variables with significant variance and related to these samples. Inspection of the HCA heat-map in Fig. 3 indicated that *P. capillacea* samples showed enriched presence of dimethoxy-luteolin-glucoside, 1-palmitoyl-GPA (16:0), carnosic acid, taxifolin, cysteine sulfinic acid in addition to caffeoylglycerol whereas a raised content of betulinic acid, nonadecenoic acid, nonadecanoic acid, kaempferol-O-pentose, glutamylglycine*,* hydroxymyristic acid and apigenin were detected in *S. hornschuchii* samples. In contrast, the second group of MS signals assigned for 6-phosphogluconate, succinic acid, echinenone, quercetin dimethyl ether, glutamylcysteine, stearidonic acid, naringenin pentose, rosmarinic acid, pentadecenoic acid and 8-pentadecenal were abundantly present in *S. platensis* samples. Moreover, *U. fasciata* samples possessed the highest relative content of lauramide, acetylornithine, fucoxanthinol, campesterol, syringic acid, isoferulic acid 3-sulfate, caffeic acid isoprenyl ester in addition to linolenic acid as depicted in Fig. 3.

In order to make meaningful dataset patterns maximizing inter-group differences of algal samples and to better assess the secondary metabolite heterogeneity in the context of phylum type accounting for discriminatory markers in the detected metabolites profile, OPLS-DA model was secondly performed.

**Figure 4:**
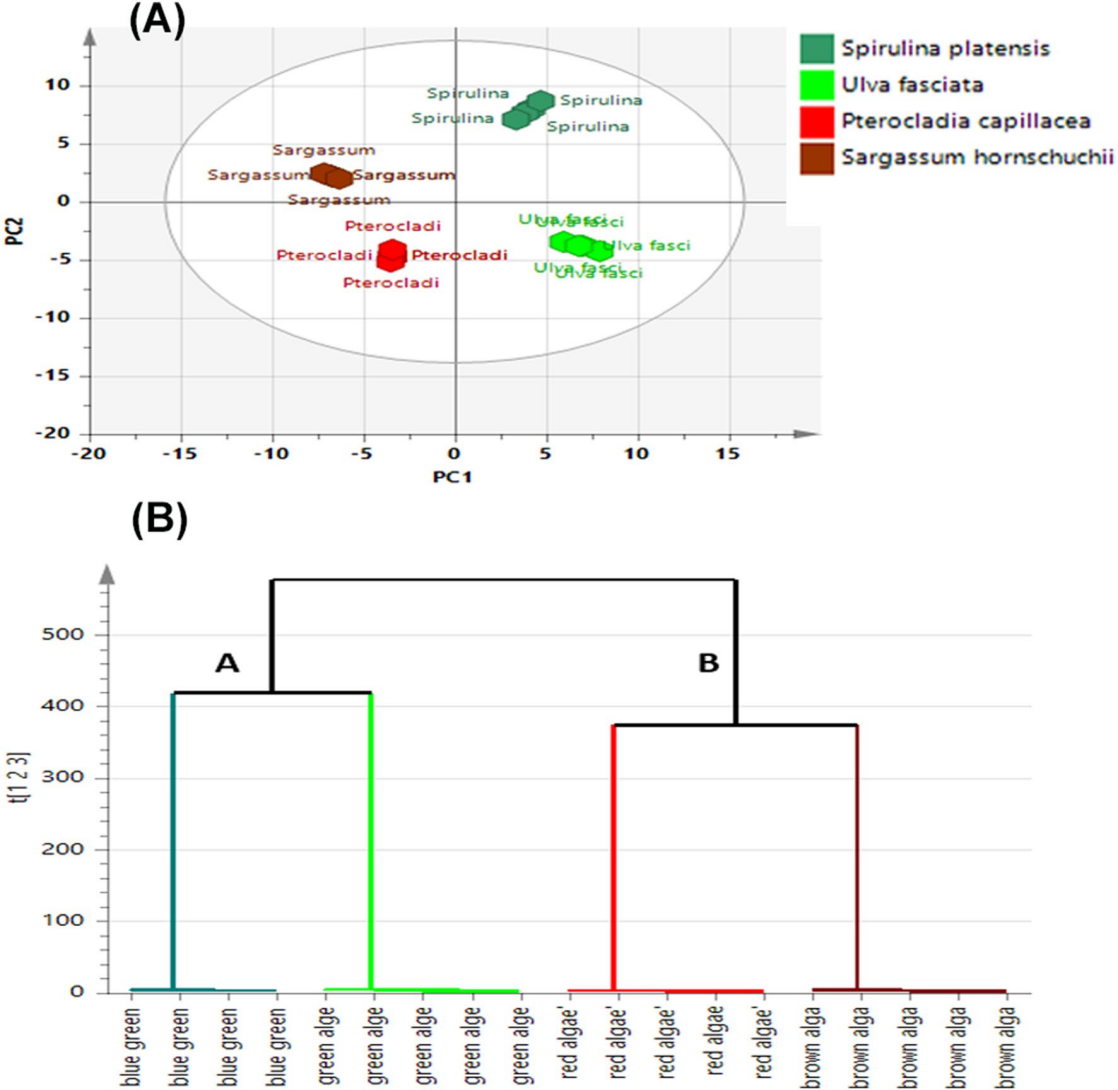
(**A**) UHPLC-MS-based OPLS-DA score plot of the algal samples, (**B**) HCA dendrogram, based on the Ward method.

OPLS-DA model (Fig. 4A) prescribed by four components effectively discriminated the four algae species into two main clusters along two orthogonal PCs, justifying 75% of the entire variance within samples. The performance of the constructed model was validated by the computed parameters “R^2^ (0.998)” and “Q^2^ (0.986)”, indicating its covered variance and good predictive capacity, respectively. Furtherly, the results of the 20 permutation tests of the OPLS-DA model were shown in Fig. S2 where interception of R^2^ and Q^2^ was less than 0.40 and 0.05, respectively, suggesting that the OPLS-DA model was not overfitted, stable and reliable. OPLS-DA Model was concerned as excellent classification model from the ROC curve presented in Fig. S3 displaying AUC value equal 1.

In agreement with the above OPLS-DA results, HCA of the four examined algae species acquired a dendrogram of two distinctly separated clusters referred to as groups **1A** and **1B**, respectively where “**1A**” group corresponding to *S. platensis* and *U. fasciata* while *S. hornschuchii* and *P. capillacea* specimens were clustered in the other group “1B” (Fig. 4B).

To rapidly capture the major characteristic metabolites discriminating between the four algal species, the corresponding OPLS-DA derived coefficient-plot, a tool that provides a better overview of the classification model metabolite markers outcome, was inspected.

**Figure 5:**
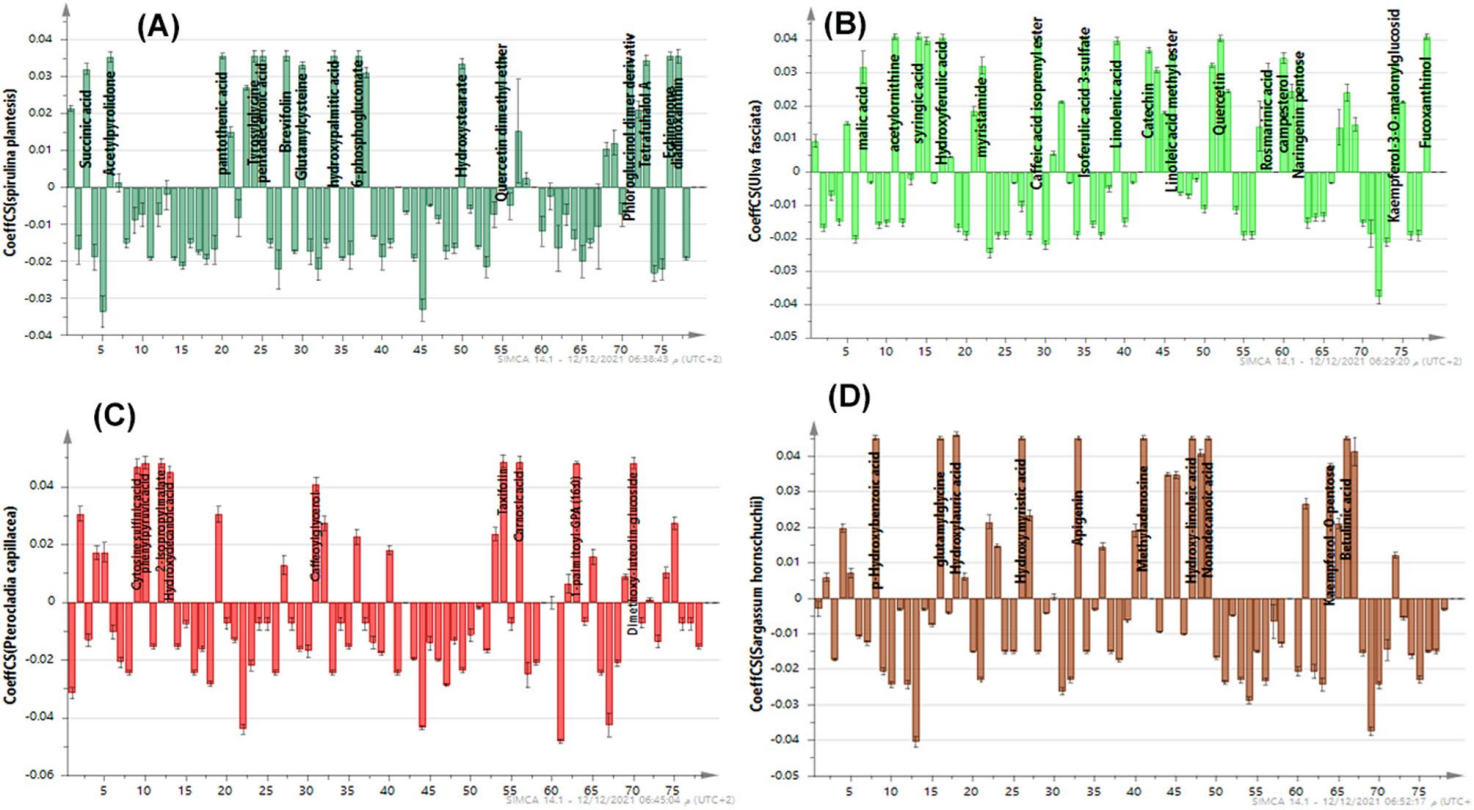
Coefficient plots of OPLS-DA model of algae species; (**A**) *S. platensis*, (**B**) *U. fasciata*, (**C**) *P. capillacea* and (**D**) *S. hornschuchii*.

As evident from the coefficient-plot (Fig. 5A). the influential metabolites primarily enriched in *S. platensis* included succinic acid, acetyl pyrrolidone, pantothenic acid, glutamylglycine, glutamylcysteine, brevifolin, 6-phosphogluconate, hydroxystearic acid, tetrafuhalol A, diadinoxanthin and echinenone. On the other hand, the notable predominant metabolites obviously detected in the coefficient-plot (Fig. 5B) namely malic acid, acetylornithine, syringic acid, hydroxyferulic acid, caffeic acid isoprenyl ester, isoferulic acid 3-sulfate, linolenic acid in addition to fucoxanthinol were differential markers enriched in *U. fasciata* samples, also explaining their distinct segregation on the lower right side of the score plot. Successively, inspection of the coefficient-plot in Fig. 5C indicated that MS peaks for cysteine sulfinic acid, phenylpyruvic acid, caffeoylglycerol, taxifolin, carnosic acid and 1-palmitoyl-GPA (16:0) served as discriminatory features abundantly present in *P. capillacea* samples clustered separately on the lower right segment of the score plot. Regarding *S. hornschuchii* samples, the coefficient-plot (Fig. 5D) showed enrichment of candidate markers such as *p*-hydroxybenzoic acid, glutamylglycine, apigenin, methyladenosine, hydroxymyristic acid, hydroxylinoleic acid, nonadecanoic acid besides betulinic acid.

### Cytotoxic activity investigation of the algal extracts and bioactive markers discovery using OPLS model

#### Cytotoxic activity of algal extracts

Cytotoxic activity of the four marine algae harvested from coastal Abu-Qir Alexandria ecosystem was determined on human prostate cancer PC3 cell line and triple negative, highly proliferative and invasive MDA-MB-231 human breast cancer cell line using the MTT assay.

**Figure 6:**
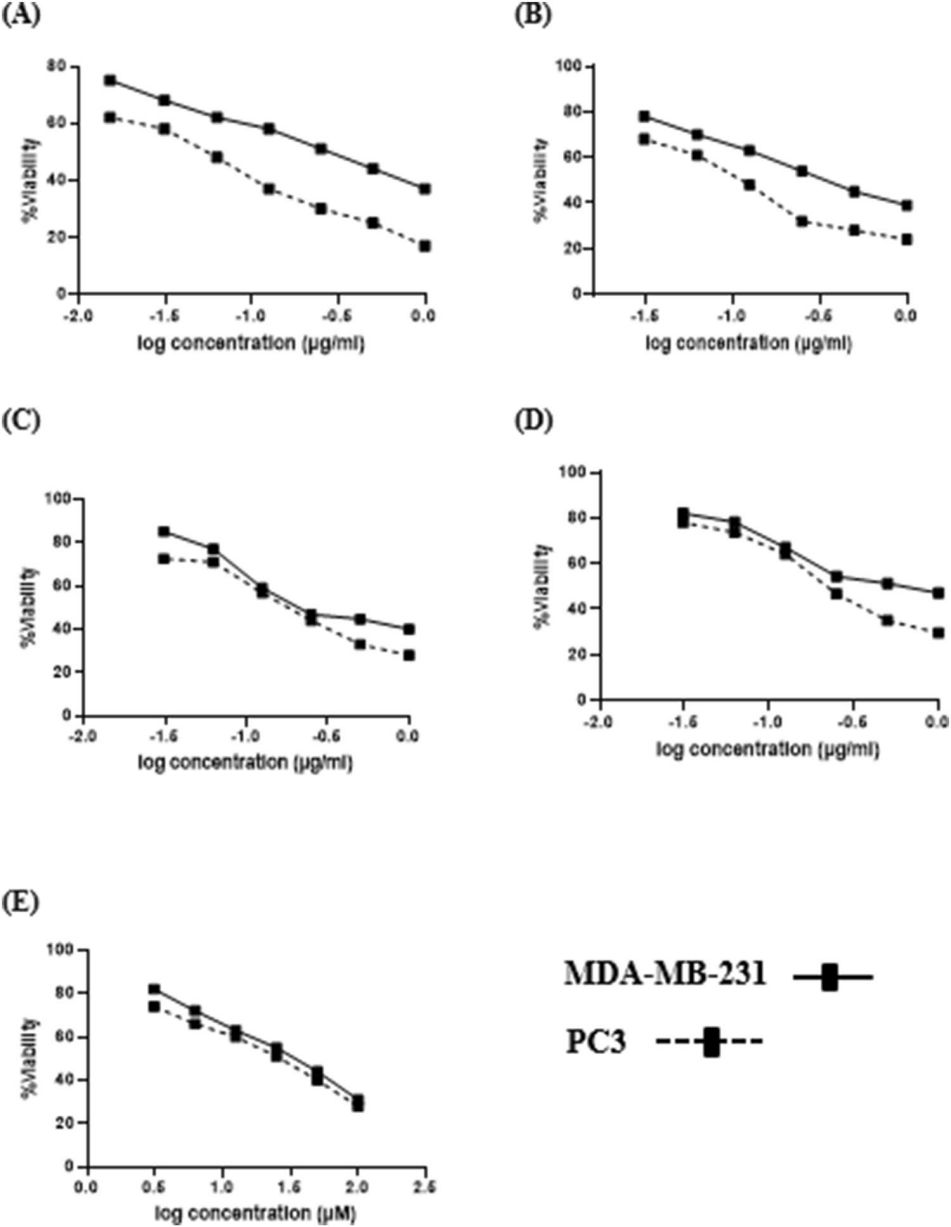
Cell viability of two cell types (PC3 (dotted lines) and MDA-MB-231 (solid lines)) exposed to different concentrations of marine algal extracts estimated by MTT assay. (**A–E**) Represent *S. platensis, U. fasciata*, *S. hornschuchii*, *P. capillacea* and 5-Fluorouracil, respectively.

Obtained results of cytotoxicity toward PC3 and MDA-MB-231 cells are presented as percentage of cell viabilities after 24 h and compared with untreated (control cells) under the same conditions (Fig. 6). Also, average inhibitory concentration capable of promoting 50% of the maximum effect (IC_50_) of the crude algal extracts against the two tumor cell lines was used to express the potential anticancer activity (Fig. 6).

To better understand the potential of algal species as essential sources of anticancer compounds, we compared their anti-proliferative effects with anticancer drug (5-Fluorouracil) already on the market and displayed relatively high potency on the two cell lines investigated (PC3 and MDA-MB-231), with IC_50_ value of 14.43 ± 1.8 and 33.6 ± 2.41 µM, respectively (Fig. 6).

The results of the present investigation evidently demonstrated that 70% ethanol extract of the four algae samples mentioned above within tested concentration range for the time of 24 h powerfully reduced PC3 and MDA-MB-231 cells survival in a dose dependent manner as presented in Fig. 6 and Table S4.

It is noteworthy to point out that a concentration of 18.45 ± 2.09 μg/mL of *S. platensis* was enough to cause cell inhibition against PC3 cell line by 50%. Successively, the response of PC3 cells exposed to *U. fasciata*, *S. hornschuchii* and *P. capillacea* extracts followed the same trend displaying significant dose-dependent decrease of PC3 cell viability with IC_50_ equal 108.12 ± 12.4, 162.56 ± 11.2 and 242.4 ± 16.3 μg/mL, respectively.

Altogether, after 24 h treatment of MDA-MB-231, a representative of highly aggressive breast cancer subtype which more likely to metastasize, all examined algal extracts induced a significant reduction in cell survival at the maximum concentration (1000 µg/mL). Data illustrated in Fig. 6 revealed a dose-dependent significant decrease in MDA-MB-231cell viability upon exposure with the microalgal *S. platensis* extract with IC_50_ equal 182.5 ± 8.6 µg/mL while *U. fasciata* and *S. hornschuchii* extracts exerted similar moderate cytotoxic activities against MDA-MB-231 with mean IC_50_ values of 320.2 ± 9.4 μg/mL and 344.3 ± 11.34 μg/mL, respectively. *P. capillacea* had relatively low potency on MDA-MB-231cell with a bit increase in its IC_50_ value; 445.21 ± 16.53 μg/mL.

Blue green algae are one of the most valuable aquatic species that presently gained increasing acclaim for its unparalleled anti-carcinogenesis due to its enrichment of diverse high-value metabolites like lipids with various structure, terpenoids, phlorotannins, various phytopigments (carotenoid and phycocyanin) and other phenolic compounds with very proven high cytotoxic capacity^22^. The anticancer capacity of *S. platensis* extracts was previously reported for some cell lines including K562, HCT116 colon carcinoma cells, and HepG2 cells^23^.

Similarly, *U. fasciata* enriched with antitumoral biomolecules induced a promising reduction in PC3 and MDA-MB-231 cells survival as a consequence of the possible joint action among its constituents.

### Efficacy directed discrimination of the different algae species and bioactive markers discovery using OPLS modeling

An OPLS model was constructed by merging the information of the peaks of MS spectra as X variables and the cytotoxic activities as Y variables to evidently mark cytotoxic discriminatory metabolites among the tested algae species. To assess for the performance of the UPLC-MS based OPLS model, calculation of the R_2_Y (explained variance) that was found to be 0.991, and Q^2^ (predicted variance) assigned as 0.982, affirming the reliability of the conducted model. Also, the results of the 20 permutation tests are shown in Fig. S4 and further proved the model was stable and reliable.

**Figure 7:**
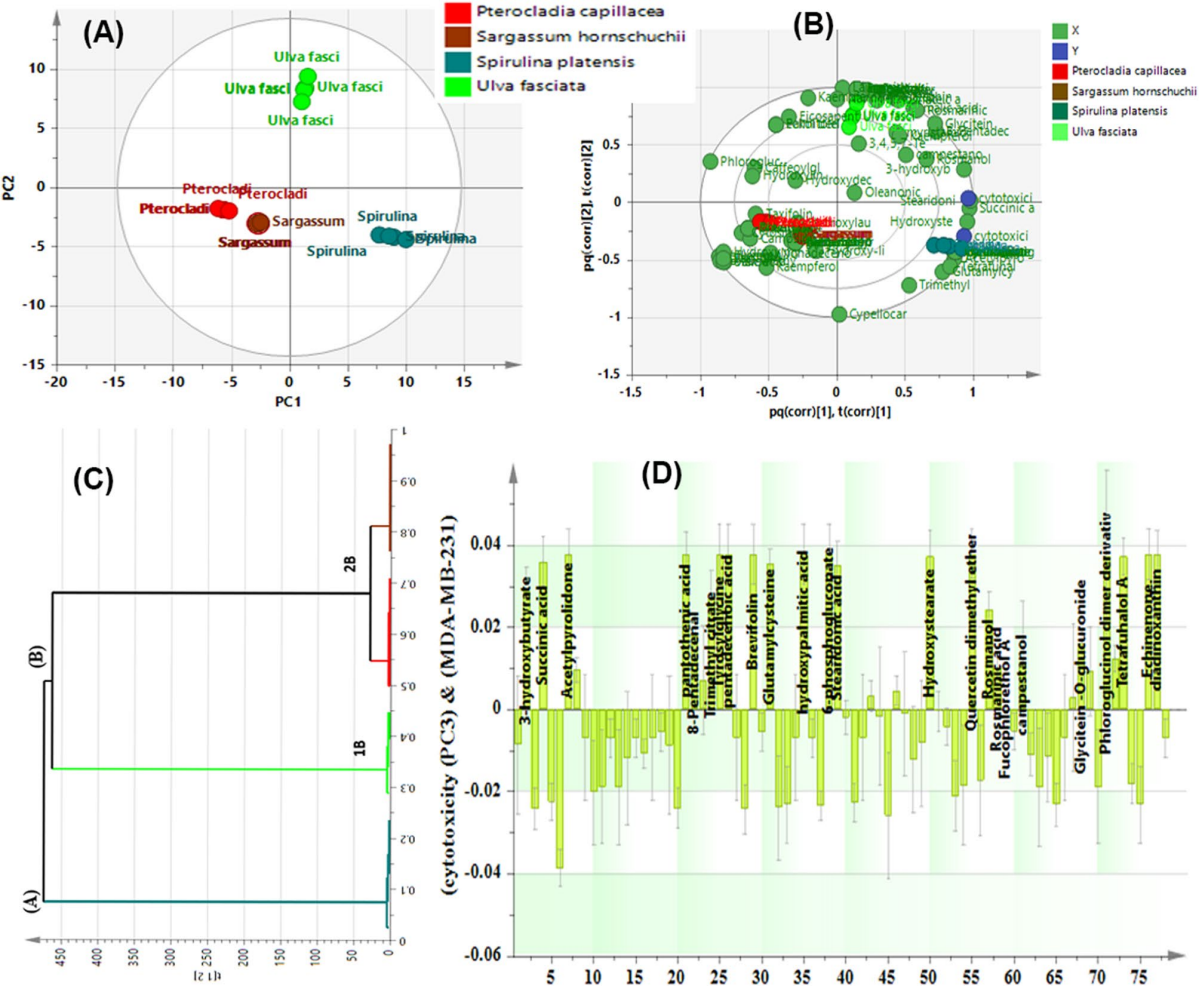
(**A**) OPLS scores plot for discrimination of algal samples according to the cytotoxic activity, (**B**) OPLS biplot representing the tested algal samples in correlation to the cytotoxicity markers, (**C**) HCA dendrogram. (**D**) Coefficient’s plot of OPLS model revealing putative bioactive markers correlated with the investigated cytotoxicity.

The results of the OPLS demonstrating 72.5% of the total variance, as represented by the obtained score plot shown in Fig. 7A effectively discriminated *S. platensis* and *U. fasciata* samples which were both clustered along the positive side of PC1 distantly from other algae samples; *S. hornschuchii* and *P. capillacea* displaying negative values for PC1 and PC2 as they were segregated in the lower left quadrant reflecting the similarity in their cytotoxicity activity. Meanwhile, PC2 significantly discriminated between *S. platensis* and *U. fasciata* samples, the latter exhibited positive values of PC2 and were positioned in the upper right quadrant whilst *S. platensis* samples revealing relatively remarkable cancerous cell proliferation inhibitory effect were readily clustered in the lower right quadrant most distantly from other tested samples (Fig. 7A). Furtherly, the OPLS-derived biplot (Fig. 7B) evidently demonstrating the spatial correlation between different algae samples and the tested antitumor activity offered a better overview of efficacy-based discrimination and further highlighted putative biomarkers directly related to remarkable bioactivities.

HCA analysis (Fig. 7C) results came in line with OPLS results as it unveiled two clear clusters referred to as groups A and B, respectively. Inspection of group A revealed that the most biologically active samples; *S. platensis* were the most distant species in comparison to others evidently clustered in one separate group B. Group B in turn displayed a distinct subcluster “1B” of *U. fasciata* samples that were almost chemically and biologically different from the rest of *S. hornschuchii* and *P. capillacea* samples grouped together in another subcluster “2B” reflecting the similarity in their cytotoxic activities.

In an attempt to deepen our knowledge about the potentially biochemical metabolites significantly connected with the measured cytotoxic activities, the coefficients plot, a dimensionless quantity tool rapidly representing the strength of the putative linear association between the variables was furtherly implemented where the variables with positive and higher magnitude are considered the significant efficacy-related contributors.

The coefficient plot obtained by OPLS model and represented in Fig. 7D revealed that plentiful chemically diverse metabolites largely correlated with the tested cytotoxic activity.

For the sake of clarity, the abundance of fatty acids and their derivatives namely as pentadecenoic acid, hydroxypalmitic acid, stearidonic acid, hydroxy stearate and linolenic acid in *S. platensis* were ascribed to its significant cytotoxicity against PC3 and MDA-MB-231 cells. Relatedly, prior work discussed the beneficial effects of free fatty acids (saturated and unsaturated) primarily linolenic acid, stearidonic acid, docosahexaenoic and palmitic acid on the progress of carcinogenesis in different tumor cell lines as breast cancer (MCF-7/MDA-MB-231), lung cancer (A549), and hepatocarcinoma (HepG2) where the key mechanism proposed for the chemosensitization or reversing multidrug chemoresistance is based on their impact on the architecture of the cell membrane and their effect on drug uptake/efflux and transporter activity thus they can increase the efficacy of chemotherapeutic drugs^24^. Furthermore, their consumption can slow the growth of tumors, by inducing the apoptotic process in tumor cells and inhibiting angiogenesis^24^.

Furtherly, biologically active principal metabolites belonging to dipeptides and other nitrogenous compounds as glutamylglycine, glycyltyrosine and acetyl pyrrolidone found more enriched in *S. platensis* and *U. fasciata* samples with relatively marked cytotoxicity against could significantly inhibit the proliferative activity of PC3 and MDA-MB-231 tumor cells. Presently, it is acknowledged that peptides exhibited profound cytotoxic potential against different carcinomas, i.e., pancreatic, colon and cervical sarcomas by triggering cell cycle arrest representing promising anticancer therapeutic leads^25^.

Equally important, rosmanol, echinenone and diadinoxanthin abundantly present in *S. platensis* and *U. fasciata* samples were significantly associated with the observable cytotoxic potential as depicted in Fig. 7D. Indeed, a recent investigation pointed out that rosmanol inhibited the proliferation of MCF-7 and MDA-MB 231 cells via apoptotic mechanism^26^. Analogously, earlier work evidenced that some carotenoids as lutein, diadinoxanthin and lycopene exerted anti-cancer actions on lung cancer cells (NCI-H226) and MCF-7 via promotion of cell cycle arrest and attenuation of tumor angiogenesis^27^.

Additionally, the coefficient plot results (Fig. 7D) showed that the biologically active samples were particularly enriched in some influential phenolic metabolites like quercetin dimethyl ether, glycitein 7-O-glucuronide, tetrafuhalol A, phloroglucinol dimer derivative in addition to rosmarinic acid which positively mediate antiproliferative effects toward PC3 and MDA-MB-231 cells.

Importantly, it was noted that polyphenolic-rich algal extracts might be considerably effective in the suppression of cancer cell proliferation and these results came in line with previous studies that proved that polyphenolic compounds showed metabolic inhibition of xenobiotic-metabolizing enzymes disrupting cell division and colony formation^25^.

Furtherly, different species of *Ecklonia* revealed prominent anticancer properties owing to the presence of variable phlorotannin compounds as fucodiphlorethol G, eckol, dieckol, and phlorofucofuroeckol A^4^.

Considering the results of OPLS analysis, we could hypothesize that observed prominent cytotoxic effects in this study could be interpreted by the ability of all detected metabolites to act synergistically.

The study in hand offered the first attempt to discriminate the different algae species based on their efficacy-related holistic chemical profile providing valuable insight into the extraordinary potential for sourcing astounding cancer leading molecules from diverse marine algae species the first to be investigated in detail chemically and biologically. The next logical step would be to isolate exact bioactive markers in these complex extracts using various isolation means in parallel to biological testing of isolated chemicals to be more conclusive expanding effective therapeutic approaches.

## Conclusion

In summary, the study in hand offered the first systematic broad-scale metabolomics investigation of different algae species aiming to provide chemically based evidence about their exceptionally remarkable biological potentials. Results gathered in the present study pointed out the promising cytotoxic activity of the algae extracts in general, *S. platensis* in particular toward PC3 and MDA-MB-231 tumor cells. UPLC-MS-based metabolomics approach integrated with multivariate statistical analysis as well as cytotoxic activity testing rapidly uncover the compositional heterogeneity in metabolite profiles of algae extracts in the context of phylum type followed by further discrimination of the samples according to the targeted bioactivity pinpointing the most relevant chemical biomarkers which in turn signifies the importance of marine algae as a source of natural leads. OPLS analysis revealed that unsaturated fatty acids, terpenoids, dipeptides along with polyphenolic compounds being abundantly present in *S. platensis* were the significant contributors synergistically meditating its prominent anti-proliferative activity. In this context*, S. platensis* extract, an outstanding store of bioactive molecules, appears to be an extremely important oceanic candidate for future investigations aimed to explore a great scope chemically and biologically.

## Materials and methods

### Chemicals and reagents

Methanol, formic acid and dimethyl sulfoxide (DMSO) were procured from Fisher Scientific, UK. Ultra-pure water produced by a Milli-Q system was used for UPLC analysis. The reference standards: *p*-hydroxybenzoic acid, catechin and linoleic acid were purchased from Sigma-Aldrich (St. Louis, MO, USA). Dulbecco’s modified eagle medium (DMEM), 3-(4,5-dimethylthiazol-2-yl)-2,5-diphenyltetrazolium bromide (MTT), phosphate buffered saline (PBS) and 5-flourouracil were from Sigma-Aldrish (St. Louis, Mo, USA).

### Sample collection and identification

The seaweeds *Ulva fasciata* Delile (Chlorophyta), *Pterocladia capillacea* (S.G.Gmelin) (Rhodophyta), *Sargassum hornschuchii* C. Agardh (Phaeophyta) along with a well-known cyanobacterium, *Spirulina platensis* Gomont (synonym *Arthrospira platensis* Gomont) utilized in this work were collected in summer 2020 from the coastal area of Abu-Qir Alexandria-North Egypt. All samples were quickly rinsed in cold fresh water to remove any visible adhering contaminants like sand, surface salts and epiphytes. Using a light microscope, initial morphological and anatomical structures in the algal samples were examined. Based on the characteristics keys in the taxonomic publications^28,29^ and comparison with voucher specimens, the samples were taxonomically identified and further authenticated by Dr. Soad Mohy El-Din, Professor of Botany at Botany Department, Faculty of Science, Alexandria University, to whom the authors are very indebted. Algal samples were freeze-dried (Edwards High Vacuum Lyophilizer, ModE2MB, Brazil), powdered using electric blender, sieved (≤ 1 mm), accurately weighed and stored in a freezer at − 20 °C until further experiments.

### Preparation of algal samples extracts

According to previously reported optimized procedure for extraction^30^, 25 g of each lyophilized algal powders were separately submerged in 100 mL of 70% ethanol in an ultrasonic bath apparatus (3L Alpha Plus, Japan) at 35 °C for 60 min. This extraction procedure was repeated three more times and the extracts were combined. The hydroalcholic extracts were concentrated to dryness under reduced pressure using rotary evaporator at 40 °C yielding solid residues ready for use.

### Analysis of the algal extracts by ultra-high performance liquid chromatography coupled to mass spectrometry (UPLC-MS)

#### Sample and standards preparation for UPLC-MS analysis

Accurate weights (1 mg/mL) from each dried algal extract were separately prepared HPLC-grade methanol, filtered through a 0.2 μm pore size membrane (Millipore) and degassed by sonication before injection. Full loop injection volume (10 μL) of each sample was applied onto the chromatographic column. Detailed preparation of standard solutions for UPLC–MS quantification can be found under Supplementary Material.

#### UPLC conditions

Metabolic profiling marine algal species was performed on an UPLC XEVO TQD triple quadruple instrument Waters Corporation, Milford, MA01757 U.S.A. The UPLC system consisted of a Waters Acquity QSM pump, a LC-2040 (Waters) autosampler, an on-line degasser and Waters Acquity CM detector**.** Chromatographic separation was conducted using a Waters Acquity UPLC BEH C18 column (50 mm × 2.1 mm ID × 1.7 μm particle size) maintained at 30 °C. In order to broaden the elution window for the analysis of multiple classes of compounds with a wide range of polarities, the binary mobile phase consisted of acidified ultrapure water (0.1% formic acid) (Phase A) and acidified methanol (0.1% formic acid) (Phase B) was gradient eluted at a flow rate of 0.2 mL/min and programmed as follows: 0.0–2.0 min, 10% B; 2.0–5.0 min, 30% B; 5.0–15.0 min, 70% B; 15.0–25.0 min, 90% B; 25.0–30.0 min, 100% B followed by 5 min for re-equilibration**.**

### ESI–MS conditions

Eluted compounds were detected from full range acquisition covering 100–1000 m/z using triple quadrupole (TQD) mass spectrometer equipped with an electrospray ion source operating in negative and positive ion modes.

The suitably chosen set of operational conditions for ESI interface were as follows: capillary voltage of 3 kV, cone voltage; 35 V, the ion source temperature was 150 °C, the nebulizer (nitrogen gas) pressure was 35 psi, drying and sheath gas (N_2_) temperature was 440 °C and 350 °C, respectively. The drying and sheath gas flows were applied at 900 L/h and 50 L/h, respectively. The analytical run time was extended to 30 min. for auto-MS/MS, the analyte ions of interest frequently precursor ions were selectively monitored by the first quadrupole (Q1) and then dissociated at collisional energies ramped from 30 to 70 eV using nitrogen gas as a collision gas in the second variable potential quadrupole collision cell (Q2) producing relatively stable and reproducible fragmentation patterns rendered as a compound’s spectral signature. This process is denoted as collision-induced dissociation (CID). Finally, the diagnostic products ions uniquely derived from the targeted mass-selected ions were subsequently monitored in the third quadrupole mass analyzer (Q3). Finally, the detected signals of interest were recorded as an ion chromatogram for the precursor-fragment ion pair. In MS^n^ experiments, other chromatographic and mass spectrometry conditions were as previously described. UPLC-MS data pre-processing steps are given under Supplementary Material.

### UHPLC–MS metabolites characterization

Metabolite assignments were established according to retention times relative to external standards, tandem mass spectra (quasi-molecular ions as well as diagnostic MS/MS fragmentation profiles) combined with surveying reference literature data and dictionary of marine natural products database which provide open-access knowledge basis to the structure information of a huge diversity of marine-derived compounds to present high confidence level of annotation.

### Cytotoxicity assay

#### Cell lines and culture conditions

Human triple negative breast cancer (MDA-MB-231) cell lines and prostate carcinoma cell line (PC3) were obtained from obtained from National Cancer Institute (NCI), Cairo-Egypt. The cancer cells were maintained as exponentially growing cultures in DMEM media supplemented with 10% (v/v) heat deactivated FBS (Fetal Bovine Serum) (Sigma, USA), antibiotics (Penicillin 100 U/mL, Streptomycin 10 µg/mL (Sigma, USA)) and 1 mM sodium pyruvate under standard conditions (37 °C, 5% CO2) in a measured humidified atmosphere.

#### MTT cytotoxicity assay

MTT assay was conducted to determine the cytotoxic potential of the algal extracts against tumor cell lines^31^. Details of the procedure can be found under Supplementary Material.

#### Multivariate data analysis of UPLC-MS profiles

Multivariate statistical analyses were performed on the MS-scaled data using SIMCA-P version 14.0 software (Umetrics, Sweden). Initially, principal component analysis (PCA) was successfully conducted on the X-data (UPLC-MS) to gain basic insights into the general clustering pattern, trends and/or outliers among all studied algal samples through the respective score plot. Subsequently, hierarchical cluster analysis heat map was further implemented via importing MS data to Metaboanalyst 4.0 (http://www.metaboanalyst.ca) to visually present better mapping of the entire set of samples as well as explore intrinsically weighing variables with significant variance and related to these samples. Meanwhile, supervised forms of discriminant analysis like orthogonal projection to latent structures analysis discriminant analysis (OPLS-DA) was secondly performed with a view to rapidly uncover the compositional heterogeneity in metabolite profiles of algae extracts in the context of phylum type. Complementary, a biologically relevant classification model, orthogonal projection to latent structures analysis (OPLS) model was conducted to evidently screen the chief biologically relevant markers that were directly connected with the remarkable cytotoxic potential via the coefficients plot. Models performance was judged by monitoring R^2^ and Q^2^ values, where R^2^ measures the goodness of model fit, while Q^2^ is indicative of the degree of the model predictability. As well, the supervised models for quality processing discrimination were validated through response permutation testing excluding potential overfitting. Receiver operating characteristic curve (ROC) was further performed for OPLS-DA model as a validation criterion to demonstrate the model predictive ability. A successful classification model is characterized by a ROC curve with an AUC close to 1.

### Statistical analysis

GraphPad Prism *v*8 statistical software (GraphPad Software, San Diego, CA, USA) was used to analyze the data. Student t-test was performed to determine significant differences between means using IBM SPSS software package version 20.0*.* (Armonk, NY: IBM Corp). Differences at P < 0.05 were considered significant.

## Supplementary Information

Supplementary Information.
