# Supplementary material for: Comprehensive metabolomics unveil the discriminatory metabolites of some Mediterranean Sea marine algae in relation to their cytotoxic activities
Source: Sci Rep. 2022 May 16;12:8094. doi: 10.1038/s41598-022-12265-7 (PMC9110716; doi:10.1038/s41598-022-12265-7)
Supplement: Supplementary file 1 — Supplementary Information. [file 41598_2022_12265_MOESM1_ESM.docx]

**MTT cytotoxicity assay**

MTT assay was conducted to determine the cytotoxic potential of the algal extracts against tumor cell lines (Mosmann, 1983). The cancerous cell lines were seeded in the 96-well flat-bottom microtiter plate at a density of approximate 2 × 10^4^ cells/well in DMEM medium supplemented with 10 % FBS and were subsequently incubated overnight at 37 °C in a 5 % CO_2_ humid incubator. DMSO was used to prepare the master stock solutions of the algal extracts (100 mg/ml) which were then diluted with cell culture medium to freshly obtain the required working concentrations (1000, 500, 250, 125, 62.5 and 31.25 µg/ml). A volume of 50 µL of each concentration of the tested algal extracts was added to the selected cells in triplicates. 50 mL of the cell suspension without any tested treatment spiked with 50 mL of their respective culture medium and contained DMSO (0.5%) was utilized as negative control while docetaxel (10 mM) was served as positive control. The cells were then incubated for 72 hours at 37 °C. Following incubation, 10 µL of the MTT labeling reagent (5mg mL^-1^) in phosphate buffered saline (PBS) was added to each well and the plates were incubated at 37° C for 4 hours. At the end of the incubation period, the medium was removed and 100 µL of solubilizing DMSO (culture grade) was added to each well to dissolve formazan crystals formed. The cells viability was estimated by measuring absorbance at 490 nm using a Quant ELISA plate reader (Bio-tek Instruments, USA). The viability of the treated cells with various concentrations of the investigated algal extracts was normalized to the viability of the corresponding untreated (control) cells. The mean extract concentration required to inhibit cell growth by 50 % (IC_50_; the median growth inhibitory concentration) compared with a vehicle-treated control was assigned by nonlinear regression analysis of the logarithm of concentration as a function of the normalized response using the software [Prism](https://07101qqaf-1105-y-https-www-sciencedirect-com.mplbci.ekb.eg/topics/chemistry/prismatic-crystal) 8.0 (GraphPad Software).

**Standard solutions for UPLC-MS quantification**

With a view to demonstrate the chemical variability among diﬀerent algal samples, analysis of all metabolites detected in each algae extract was conducted based on mean peak area computation using the calibration curves of corresponding standards and the result were readily presented as (mg standard equivalents/g dry algae extract). The stock external standard solution of *p*-hydroxybenzoic acid, catechin and linoleic acid was prepared as follow: an accurate weight of each standard (10 mg) was separately placed in 10-mL volumetric flask. HPLC-grade methanol was added and the solution was serially diluted to the working concentrations over the reliable range 0.0125 – 0.75 mg mL^-1^ using the same solvent. Five μL aliquots of each standard compound were applied onto the chromatographic column and the injections were performed in duplicates for different concentration levels. The standard calibration curves were established by plotting peak areas of the standards as the analytical responses against their known concentration concentrations. Parameters like linearity, limit of detection (LOD) and limit of quantiﬁcation (LOQ) were assessed based on FDA guideline on bioanalytical method validation (Kadian et al., 2016) (**Table S1**).

**UPLC-MS data pre-processing**

Herein, Native UPLC-MS were firstly converted into centroid mode data of mzML format using MSConvert tool from Proteowizard program (<http://proteowizard.sourceforge.net/>) where files were arranged in one folder set as the file source. All raw UPLC-MS data were subjected to batch molecular feature detection using MZmine 2.0 (http://mzmine.sourceforge.net/) data analysis software All signals below 10e^3^ level were then neglected. Complementarily, adjacent peak deconvolution was employed to significantly minimize the complexity of dataset by grouping proximal peaks together using a moving window of 30s in retention time. Alignment of detected feature across multiple chromatograms was implicitly conducted by applying the join aligner algorithm with 60s as tolerance window in retention time to correct undesired run-to-run variability and profoundly guarantee downstream extraction of beneficial non-redundant information (Yi et al., 2016). Finally, the resulting UPLC-MS peak list (a series of 255 recorded peaks as detailed in **Table S1**) was organized in an Excel spreadsheet (Microsoft Oﬃce Excel, 2013).

**Table S1: Linearity and sensitivity parameters for *p*-hydroxybenzoic acid, catechin and linoleic acid used as external standards**

| **Compound** | **Linearity range (mg mL^-1^)** | **Slope (a)** | **Intercept (b)** | **r** | **LOD (mg mL^-1^)** | **LOQ (mg mL^-1^)** |
| --- | --- | --- | --- | --- | --- | --- |
| ***p*-hydroxybenzoic acid** | **0.0125-0.25** | 1.55*10^8^ | -6.91*10^3^ | **0.996** | **0.009** | **0.0125** |
| **Catechin** | **0.025-0.536** | 2.43*10^8^ | -8.34*10^4^ | **0.993** | **0.011** | **0.025** |
| **Linoleic acid** | **0.03-0.6** | 1.63*10^8^ | -9.2*10^4^ | **0.994** | **0.013** | **0.03** |

**Experimental conditions as in Section 2.4.2. For each calibration curve the equation is y= ax + b, where y is the peak area, x is the concentration of the standard (mg mL^-1^), a is the slope, b is the intercept and r the correlation coefﬁcient.**

**Table S2: UPLC-MS metabolite profiling data from all algal samples representing the content of each variable (peak areas)**

| **Compound names (m/z)** | ***Spirulina platensis*** | ***Ulva fasciata*** | ***Pterocladia capillacea*** | ***Sargassum hornschuchii*** |
| --- | --- | --- | --- | --- |
| 100.2 | 0 | 272340 | 1902130 | 0 |
| 102.1 | 0 | 2134501 | 0 |  |
| malonic acid | 581308 | 986560 | 609159 | 849814 |
| 103.23 | 909646 | 198656 | 708900 | 0 |
| 104.2 | 0 | 0 | 987123 | 6541231 |
| 3-hydroxybutyrate | 695357 | 558738 | 0 | 267073 |
| 105.2 | 823876 | 0 | 564877 | 0 |
| 106.2 | 1765421 | 0 | 0 | 7861234 |
| 107.2 | 254600 | 0 | 0 | 0 |
| 107.22 | 267412 | 0 | 0 | 0 |
| 111.23 | 0 | 0 | 0 | 884626 |
| 111.2 | 885142 | 1558738 | 523398 | 0 |
| 113.1 | 288514 | 1258738 | 323398 | 0 |
| 113.2 | 568519 | 758738 | 0 | 0 |
| 113.2 | 378514 | 958738 | 0 | 0 |
| (glycerol) 114.75 | 0 | 0 | 6176518 | 4176518 |
| 115.2 | 3182000 | 0 | 0 | 0 |
| 116.2 | 0 | 1156310 | 0 | 0 |
| Succinic acid | 3309870 | 1186560 | 0 | 0 |
| 117.12 | 0 | 0 | 0 | 6759801 |
| 117.24 | 0 | 0 | 0 | 6259802 |
| 117.3 | 0 | 345601 | 0 | 0 |
| Valine | 0 | 0 | 5191624 | 0 |
| 120.14 | 0 | 2748180 | 0 | 0 |
| 124.1 | 851098 | 0 | 5671231 | 0 |
| Phloroglucinol | 0 | 7268072 | 9079438 | 6862911 |
| 127.3 | 0 | 0 | 0 | 1495449 |
| Acetylpyrolidone | 43223011 | 0 | 0 | 5236793 |
| 129.34 | 0 | 0 | 3396473 | 0 |
| 132.2 | 1654901 | 295643 | 0 | 0 |
| 132.24 | 0 | 0 | 4726584 | 0 |
| malic acid | 2586407 | 5268072 | 0 | 0 |
| 133.24 | 0 | 0 | 0 | 4831083 |
| 135.1 | 3385586 | 5470677 | 5526042 | 0 |
| 135.2 | 0 | 0 | 0 | 3894415 |
| 135.24 | 378900 | 387654 | 345678 | 0 |
| 136.2 | 567530 | 0 | 0 | 0 |
| 137.22 | 56700 | 765800 | 0 | 34560 |
| p-Hydroxybenzoic acid | 0 | 0 | 0 | 1613657 |
| 138.33 | 0 | 0 | 6326275 | 0 |
| 139.1 | 0 | 0 | 0 | 530000 |
| 139.23 | 0 | 0 | 0 | 5285152 |
| 140.12 | 2786101 | 0 | 0 | 0 |
| 142.2 | 0 | 0 | 0 | 2412310 |
| 143.2 | 3450981 | 3356432 | 3125411 | 2412310 |
| 147.1 | 1200895 | 5331373 | 4878933 | 1988997 |
| 147.2 | 0 | 0 | 0 | 2096791 |
| Hydroxyglutaric acid | 0 | 0 | 1523460 | 7500000 |
| 148.23 | 572021 | 496949 | 2269837 | 594654 |
| 149.2 | 476520 | 5976542 | 3412351 | 2134110 |
| 151.2 | 0 | 1452310 | 0 | 0 |
| Cytosine sulfinic acid | 1202270 | 1573565 | 2635364 | 30823 |
| 153.2 | 0 | 1246123 | 0 | 0 |
| 154.2 | 0 | 0 | 2123451 | 0 |
| 156.1 | 2064675 | 1101898 | 6294835 | 510489 |
| 156.2 | 547097 | 629483 | 484228 | 626380 |
| 157.2 | 0 | 103233 | 7853212 | 612439 |
| 157.2 | 0 | 725000 | 654879 | 765099 |
| 157.3 | 4202270 | 573565 | 0 | 0 |
| 158.12 | 0 | 291723 | 6071813 | 0 |
| Phenylpyruvic acid | 0 | 0 | 5523413 | 0 |
| 165.2 | 0 | 0 | 0 | 6959512 |
| 169.3 | 0 | 2912341 | 0 | 1012013 |
| 171.11 | 0 | 0 | 0 | 2121814 |
| Acetylornithine | 0 | 3830257 | 0 | 0 |
| 174.21 | 0 | 0 | 1963185 | 451678 |
| 175.11 | 0 | 0 | 9340460 | 134864 |
| 2-Isopropylmalate | 0 | 0 | 2511295 | 0 |
| 177.23 | 0 | 0 | 0 | 151264 |
| 178.2 | 856000 | 0 | 0 | 0 |
| 180.2 | 1785400 | 0 | 0 | 5123100 |
| 185.3 | 186870 | 0 | 0 | 0 |
| Hydroxydecanoic acid | 1815796 | 2410427 | 4133392 | 342710 |
| 192.2 | 0 | 0 | 5123410 | 3765123 |
| 195.2 | 2456700 | 4561231 | 761231 | 142356 |
| syringic acid | 0 | 13381148 | 0 | 0 |
| 198.2 | 256009 | 0 | 0 | 0 |
| 199.2 | 0 | 131884 | 4529056 | 0 |
| 200.1 | 204168 | 0 | 0 | 840053 |
| Lauramide | 0 | 52604561 | 18817614 | 0 |
| 201.3 | 1429977 | 771234 | 0 | 0 |
| 202.3 | 1598785 | 1805813 | 0 | 650384 |
| glutamylglycine | 12505527 | 0 | 0 | 11509527 |
| Methyl citrate | 5504231 | 404231 | 0 | 0 |
| 208.12 | 0 | 805813 | 0 | 5840491 |
| Hydroxyferulic acid | 504231 | 9909588 | 0 | 0 |
| 210.2 | 387600 | 456123 | 2812341 | 2161100 |
| 211.2 | 654320 | 1891234 | 2134521 | 5412310 |
| 214.2 | 0 | 891234 | 4146551 | 0 |
| Hydroxylauric acid | 0 | 2575146 | 0 | 13582710 |
| 215.19 | 0 | 543490 | 4603958 | 954500 |
| 216.11 | 0 | 0 | 0 | 7642904 |
| 216.24 | 95123 | 291234 | 0 | 764290 |
| glycerin triacetate | 0 | 0 | 31218123 | 21218209 |
| pantothenic acid | 1642751 | 0 | 0 | 0 |
| 222.12 | 0 | 1712341 | 1312410 | 1234121 |
| 223.14 | 0 | 7156133 | 0 | 0 |
| 8-Pentadecenal | 20138156 | 22267414 | 10088913 | 9060918 |
| myristamide | 203199401 | 281655657 | 210961458 | 306501888 |
| 229.12 | 571039 | 2102539 | 0 | 0 |
| 231.22 | 0 | 0 | 1146554 | 0 |
| Trimethyl citrate | 26499440 | 0 | 2274501 | 20887043 |
| 235.23 | 0 | 0 | 0 | 7557319 |
| 236.12 | 230063 | 299355 | 0 | 0 |
| Glycyltyrosine | 40977154 | 0 | 0 | 0 |
| pentadecenoic acid | 7002121 | 0 | 0 | 0 |
| 240.1 | 0 | 716768 | 0 | 399377 |
| 242.2 | 0 | 223412 | 0 | 2312310 |
| Hydroxymyristic acid | 0 | 0 | 0 | 7533468 |
| Hydroxy myristamide | 9285991 | 11275530 | 22913865 | 25867028 |
| 244.5 | 0 | 0 | 5801941 | 5920762 |
| Brevifolin | 5015774 | 0 | 0 | 0 |
| Caffeic acid isoprenyl ester | 383364 | 7293362 | 0 | 0 |
| 249.23 | 916812 | 8922532 | 7635788 | 0 |
| 250.12 | 0 | 623817 | 215123 | 0 |
| Glutamylcysteine | 1562135 | 0 | 0 | 618396 |
| 252.12 | 0 | 0 | 0 | 520598 |
| Caffeoylglycerol | 0 | 2561231 | 4998609 | 0 |
| 255.2 | 0 | 0 | 765432 | 0 |
| 256.12 | 229730 | 884618 | 0 | 2185397 |
| 257.22 | 1960788 | 537370 | 0 | 250203 |
| 258.12 | 465852 | 5975923 | 0 | 5313430 |
| 259.2 | 245678 | 2765123 | 261231 | 0 |
| 261.2 | 345621 | 161234 | 0 | 1543218 |
| 263.23 | 0 | 0 | 0 | 2371396 |
| 266.2 | 2345001 | 9712341 | 2612310 | 5612311 |
| Palmitoleic acid methyl ester | 0 | 7909589 | 9909588 | 0 |
| Apigenin | 0 | 0 | 0 | 5937829 |
| 270.15 | 483033 | 0 | 0 | 0 |
| hydroxypalmitic acid | 10421565 | 0 | 0 | 0 |
| Isoferulic acid 3-sulfate | 0 | 13972859 | 0 | 0 |
| 3’-O-Methylequol | 0 | 0 | 0 | 9046718 |
| 6-phosphogluconate | 29798460 | 0 | 0 | 0 |
| Stearidonic acid | 4123513 | 1723100 | 0 | 0 |
| Linolenic acid | 26262178 | 165553367 | 0 | 0 |
| 280.12 | 0 | 0 | 0 | 835343 |
| oleic acid | 0 | 0 | 1147087 | 4221726 |
| 281.2 | 0 | 955720 | 0 | 0 |
| Methyladenosine | 0 | 0 | 0 | 6543206 |
| 282.2 | 0 | 940502 | 0 | 0 |
| Dihydroxypalmitic acid | 3887123 | 7995974 | 0 | 0 |
| Catechin | 27942536 | 89227235 | 0 | 0 |
| 3,4,5,7-Tetrahydroxyisoﬂavanone | 32729583 | 89227235 | 0 | 85498859 |
| 290.12 | 2456781 | 2987123 | 0 | 1623120 |
| 291.22 | 0 | 876952 | 1335157 | 0 |
| Hydroxylinolenic acid | 0 | 27155521 | 18352419 | 41699041 |
| Linoleic acid methyl ester | 1186741 | 3370438 | 0 | 0 |
| 294.14 | 203653 | 92995974 | 0 | 0 |
| Hydroxy-linoleic acid | 3768695 | 683896 | 0 | 20268826 |
| Nonadecenoic acid | 0 | 0 | 683896 | 4875482 |
| 296.12 | 427454 | 416712 | 0 | 265550 |
| Nonadecanoic acid | 0 | 537370 | 837370 | 13758504 |
| 298.12 | 0 | 0 | 1191652 | 0 |
| Methyl stearate | 4527948 | 953344 | 0 | 0 |
| Hydroxystearate | 5423664 | 4853344 | 0 | 0 |
| 4-Hydroxybenzoic acid 4-O-glucoside | 2111581 | 0 | 1034743 | 0 |
| Ellagic acid | 1873444 | 4554317 | 1637328 | 0 |
| Quercetin | 1527946 | 5975923 | 0 | 0 |
| 301.12 | 467946 | 467821 | 0 | 456789 |
| Eicosapentaenoic acid | 456280 | 7770971 | 6688390 | 50521 |
| Taxifolin | 562543 | 1034743 | 6158945 | 0 |
| 317.2 | 1003055 | 0 | 0 | 0 |
| 318.3 | 1115818 | 3788051 | 0 | 0 |
| 319.2 | 720000 | 0 | 850000 | 0 |
| 321.2 | 0 | 0 | 0 | 1198525 |
| 325.2 | 274567 |  |  |  |
| Quercetin dimethyl ether | 2860903 | 0 | 0 | 0 |
| Carnosic acid | 21115818 | 0 | 11607591 | 12060253 |
| 334.2 | 400413 | 456700 | 0 | 0 |
| 343.2 | 0 | 0 | 1923100 | 0 |
| Rosmanol | 96622404 | 96412050 | 82109822 | 86196470 |
| 353.2 | 105453 | 563087 | 1359348 | 0 |
| Rosmarinic acid | 1532524 | 1457482 | 0 | 0 |
| 363.12 | 128712 | 0 | 0 | 0 |
| 365.2 | 0 | 170155 | 0 | 0 |
| Fucophlorethol A | 2651234 | 0 | 0 | 0 |
| campesterol | 43964582 | 74359369 | 47305745 | 31592469 |
| 393.2 | 0 | 412356 | 265123 | 1623410 |
| campestanol | 27809885 | 32336124 | 15764575 | 30301250 |
| Naringenin pentose | 10554134 | 16555336 | 12597169 | 9095913 |
| 1-palmitoyl-GPA (16:0) | 0 | 0 | 649199 | 0 |
| Kaempferol -O-pentose | 836671 | 77460 | 2455404 | 0 |
| 421.4 | 5924971 | 6917890 | 5997231 | 0 |
| 422.2 | 91727 | 0 | 0 | 0 |
| 425.2 | 278098 | 3233611 | 15764575 | 8169528 |
| 433.12 | 1456700 | 1782341 | 1523411 | 6719180 |
| 441.2 | 0 | 0 | 0 | 600198 |
| 441.56 | 0 | 0 | 0 | 848524 |
| 443.2 | 0 | 0 | 0 | 675432 |
| Taxifolin-O-rhamnoside | 1741620 | 2420883 | 7486466 | 8263847 |
| 453.1 | 398761 | 0 | 112345 | 0 |
| Betulinic acid | 0 | 0 | 0 | 4256822 |
| Oleanonic acid | 25768452 | 27155521 | 22582927 | 30301250 |
| Glycitein -O-glucuronide | 255932 | 2561698 | 0 | 0 |
| Kaempferol-methylether-O-glucoside | 42708568 | 52995974 | 70782039 | 33416385 |
| 471.2 | 506861 | 671234 | 0 | 0 |
| 473.2 | 187651 | 5123461 | 1723411 | 182310 |
| Dimethoxy-luteolin-glucoside | 0 | 0 | 2657040 | 0 |
| 477.2 | 497650 | 643124 | 451342 | 0 |
| 481.2 | 176523 | 198712 | 1412356 | 0 |
| 485.2 | 0 | 0 | 0 | 2657040 |
| 499.2 | 0 | 0 | 1712341 | 6312351 |
| 507.1 | 0 | 0 | 1321451 | 0 |
| 515.1 | 1455140 | 0 | 0 | 0 |
| Phloroglucinol dimer derivative | 2377615 | 0 | 0 | 0 |
| Cypellocarpin c | 46914198 | 0 | 33927746 | 48861734 |
| 527.2 | 15052822 | 249587 | 0 | 0 |
| Tetrafuhalol A | 18052822 | 299587 | 269587 | 4790230 |
| Kaempferol-3-O-malonylglucoside | 0 | 9306690 | 4934561 | 0 |
| Eckol derivative | 0 | 1071067 | 2001067 | 0 |
| Echinenone | 4380205 | 0 | 0 | 0 |
| 560.2 | 225871 | 361234 | 0 | 0 |
| 579.2 | 143688 | 2345120 | 0 | 0 |
| diadinoxanthin | 18052822 | 0 | 0 | 0 |
| 591.2 | 0 | 0 | 4612341 | 0 |
| Fucoxanthinol | 13052822 | 3788051 | 0 | 0 |
| 637.2 | 0 | 520980 | 0 | 0 |
| 653.2 | 1876511 | 0 | 0 | 0 |
| 671.2 | 4525407 | 2986541 | 0 | 1561231 |
| 677.2 | 124565 | 1234651 | 0 | 0 |
| C18: 3/C13:0 phosphatidylglycerol | 14052822 | 0 | 0 | 0 |
| 713 | 404145 | 0 | 0 | 0 |
| 721.2 | 230003 | 0 | 0 | 0 |
| 723.1 | 265420 | 0 | 0 | 0 |
| 743.2 | 598752 | 6543210 | 4123511 | 3412360 |
| C16: 1 /C20: 5 phosphatidylglycerol | 12052822 | 0 | 0 | 0 |

*****Each peak area is the average of three determinations.

**Table S3: Content of identified metabolites in algal samples analyzed by UPLC-MS (data are expressed as mg standard equivalent per gram of dry extract)**

| **Identiﬁed compounds** | ***Spirulina platensis*** | ***Ulva fasciata*** | ***Pterocladia capillacea*** | ***Sargassum hornschuchii*** |
| --- | --- | --- | --- | --- |
| 6-phosphogluconate**** | 0.19 | 0 | 0 | 0 |
| Methylcitrate**** | 0.012 | ˂LOD | 0 | 0 |
| Glycerol **** | 0 | 0 | 0.04 | 0.027 |
| Glycerin triacetate**** | 0 | 0 | 0.2 | 0.13 |
| 3-Hydroxybutyrate**** | 0.045 | 0.04 | 0 | 0.021 |
| Malonic acid**** | 0.04 | 0.057 | 0.044 | 0.053 |
| Cysteine sulfinic acid**** | 0.011 | 0.012 | 0.022 | ˂LOD |
| 4-Hydroxybenzoic acid 4-O-glucoside**** | 0.018 | 0 | 0.01 | 0 |
| Valine**** | 0 | 0 | 0.037 | 0 |
| Malic acid**** | 0.02 | 0.032 | 0 | 0 |
| 2-Hydroxyglutaric acid**** | 0 | 0 | 0.014 | 0.052 |
| Methyladenosine**** | 0 | 0 | 0 | 0.046 |
| Succinic acid**** | 0.025 | 0.013 | 0 | 0 |
| Glutamylglycine **** | 0.085 | 0 | 0 | 0.078 |
| Isoferulic acid 3-sulfate**** | 0 | 0.094 | 0 | 0 |
| *p*-Hydroxybenzoic acid**** | 0 | 0 | 0 | 0.015 |
| Syringic acid**** | 0 | 0.09 | 0 | 0 |
| Acetyl pyrrolidone**** | 0.28 | 0 | 0 | 0.037 |
| Phloroglucinol**** | 0 | 0.018 | 0.059 | 0.044 |
| Caffeoylglycerol**** | 0 | 0.017 | 0.032 | 0 |
| Ellagic acid**** | 0.012 | 0.032 | 0.011 | 0 |
| Trimethyl citrate**** | 0.17 | 0 | 0.015 | 0.13 |
| Catechin*** | 0.12 | 0.36 | 0 | 0 |
| Acetylornithine**** | 0 | 0.025 | 0 | 0 |
| Glutamylcysteine**** | 0.011 | 0 | 0 | ˂LOD |
| Phenylpyruvic acid**** | 0 | 0 | 0.036 | 0 |
| Brevifolin**** | 0.032 | 0 | 0 | 0 |
| 2-Isopropylmalate**** | 0 | 0 | 0.014 | 0 |
| Hydroxyferulic acid**** | ˂LOD | 0.064 | 0 | 0 |
| Pantothenic acid**** | 0.011 | 0 | 0 | 0 |
| Rosmarinic acid**** | 0.01 | ˂LOD | 0 | 0 |
| 3,4,5,7-Tetrahydroxyisoﬂavanone*** | 0.14 | 0.56 | 0 | 0.5 |
| Caffeic acid isoprenyl ester**** | ˂LOD | 0.047 | 0 | 0 |
| Phloroglucinol dimer derivative**** | 0.015 | 0 | 0 | 0 |
| Glycyltyrosine**** | 0.26 | 0 | 0 | 0 |
| Kaempferol -O-pentose*** | ˂LOD | ˂LOD | 0.011 | 0 |
| 3’-O-Methylequol*** | 0 | 0 | 0 | 0.037 |
| Naringenin pentose*** | 0.043 | 0.068 | 0.052 | 0.037 |
| Kaempferol-methylether--O-glucoside*** | 0.17 | 0.19 | 0.24 | 0.16 |
| Hydroxydecanoic acid** | 0.011 | 0.016 | 0.027 | ˂LOD |
| Taxifolin-O-rhamnoside*** | ˂LOD | 0.018 | 0.03 | 0.034 |
| Rosmanol** | 0.26 | 0.25 | 0.22 | 0.23 |
| Dimethoxy-luteolin-glucoside*** | 0 | 0 | 0.011 | 0 |
| Kaempferol-3-O-malonylglucoside*** | 0 | 0.038 | 0.02 | 0 |
| Cypellocarpin C** | 0.13 | 0 | 0.14 | 0.093 |
| 8-Pentadecenal** | 0.055 | 0.06 | 0.027 | 0.028 |
| Quercetin*** | ˂LOD | 0.025 | 0 | 0 |
| Lauramide** | 0 | 0.14 | 0.052 | 0 |
| Hydroxy myristamide** | 0.025 | 0.031 | 0.063 | 0.07 |
| Oleanonic acid** | 0.07 | 0.075 | 0.062 | 0.083 |
| 1-palmitoyl-GPA (16:0) ** | 0 | 0 | ˂LOD | 0 |
| Glycitein 7-O-glucuronide*** | ˂LOD | 0.011 | 0 | 0 |
| Myristamide** | 0.55 | 0.57 | 0.7 | 0.78 |
| Taxifolin*** | ˂LOD | ˂LOD | 0.026 | 0 |
| Apigenin*** | 0 | 0 | 0 | 0.025 |
| Quercetin dimethylether*** | 0.013 | 0 | 0 | 0 |
| Fucophlorethol A**** | 0.017 | 0 | 0 | 0 |
| Hydroxylauric acid** | 0 | ˂LOD | 0 | 0.038 |
| Hydroxylinolenic acid** | 0 | 0.075 | 0.05 | 0.11 |
| Pentadecenoic acid** | 0.02 | 0 | 0 | 0 |
| C16: 1 /C20: 5 phosphatidylglycerol** | 0.033 | 0 | 0 | 0 |
| Tetrafuhalol A**** | 0.12 | ˂LOD | ˂LOD | 0.03 |
| Eckol derivative**** | 0 | ˂LOD | 0.013 | 0 |
| Carnosic acid** | 0.05 | 0 | 0.03 | 0.035 |
| Linolenic acid** | 0.08 | 0.4 | 0 | 0 |
| Stearidonic acid** | 0.012 | ˂LOD | 0 | 0 |
| Dihydroxypalmitic acid** | 0.01 | 0.022 | 0 | 0 |
| C18: 3 /C13:0 phosphatidylglycerol** | 0.09 | 0 | 0 | 0 |
| Echinenone** | 0.012 | 0 | 0 | 0 |
| Palmitoleic acid methyl ester** | 0 | 0.022 | 0.028 | 0 |
| Hydroxylinoleic acid** | 0.01 | ˂LOD | 0 | 0.056 |
| Eicosapentaenoic acid** | ˂LOD | 0.022 | 0.019 | ˂LOD |
| Fucoxanthinol** | 0.036 | 0.01 | 0 | 0 |
| Hydroxymyristic acid** | 0 | 0 | 0 | 0.021 |
| Linoleic acid methyl ester** | ˂LOD | 0.011 | 0 | 0 |
| diadinoxanthin** | 0.05 | 0 | 0 | 0 |
| Hydroxypalmitic acid** | 0.029 | 0 | 0 | 0 |
| Campesterol** | 0.12 | 0.22 | 0.13 | 0.09 |
| Hydroxystearic acid** | 0.016 | 0.015 | 0 | 0 |
| Oleic acid** | 0 | 0 | ˂LOD | 0.011 |
| Nonadecenoic acid** | 0 | 0 | ˂LOD | 0.012 |
| Nonadecanoic acid** | 0 | ˂LOD | ˂LOD | 0.038 |
| Campestanol** | 0.078 | 0.09 | 0.044 | 0.087 |
| Betulinic acid** | 0 | 0 | 0 | 0.013 |
| Methyl stearate** | 0.013 | ˂LOD | 0 | 0 |

*Data are expressed as the average of five determinations (n=5).

**Fatty acids and their derivatives as well as terpenoids were quantified linoleic acid equivalent.

***Flavonoids were quantified as catechin equivalent.

**** Phenolic and organic acids were quantified as *p*-hydroxybenzoic acid equivalent.

**Table S4: Cytotoxic effects of human breast (MDA-MB-231) and prostate (PC3) cancer cells after exposure to different concentrations of marine algal extracts as determined by MTT assay (Results were obtained from three separate experiments; mean value ± SD).**

| **Concentration**  **(μg mL^-1^)** | **%Cell viability** | | | | | | | |
| --- | --- | --- | --- | --- | --- | --- | --- | --- |
|  | ***S. platensis*** | | ***U. fasciata*** | | ***S. hornschuchii*** | | ***P. capillacea*** | |
|  | MDA-MB-231 | PC3 | MDA-MB-231 | PC3 | MDA-MB-231 | PC3 | MDA-MB-231 | PC3 |
| 1000 | 37±3.2 | 16±1.8 | 39±2.2 | 24±1.6 | 40±2.9 | 28±1.8 | 47±2.4 | 29.6±2.1 |
| 500 | 44±3.2 | 24±2.1 | 45±1.9 | 28±2.3 | 44±3.1 | 33±1.9 | 51±2.4 | 35±2.5 |
| 250 | 51±3.4 | 30±2.1 | 54±3.3 | 32±3.4 | 47±2.8 | 44±3.2 | 54.3±2.5 | 46±3.4 |
| 125 | 58±3.1 | 37±2.6 | 63±3.8 | 48±2.5 | 59±3.2 | 56±2.3 | 67±4.1 | 64±3.7 |
| 62.5 | 62±2.5 | 48±4.2 | 70±3.4 | 61±4.1 | 77±4.5 | 71±4.7 | 78.2±3.9 | 73±3.7 |
| 31.25 | 68±4.4 | 55±4.5 | 78±4.8 | 68±4.6 | 85±5.3 | 73.5±4.5 | 82±5.8 | 78±5.1 |
| 15.625 | 75±5.3 | 62±3.9 | 85±4.8 | 79±5.7 | 90±5.2 | 80.5±5.1 | 93±5.9 | 89±4.7 |

**Figure S1:** PCA score scatter plot of the tested algae samples.

**Figure S2:** The plot showing the result of permutation test for the OPLS-DA model.

**Figure S3:** ROC curve of the established OPLS-DA model


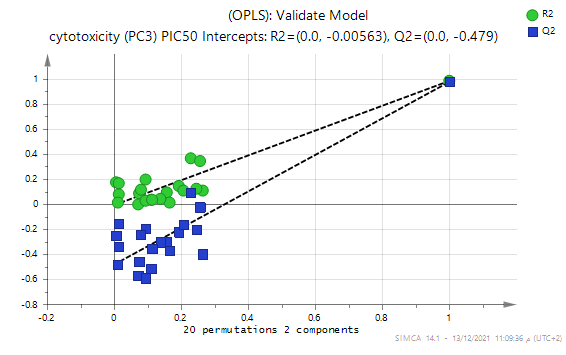


**Figure S4:** The plot showing the result of permutation test for the OPLS model.
